# Development of Efficient Covalent Inactivators of a Fungal Aspartate Semialdehyde Dehydrogenase

**DOI:** 10.1002/ddr.70095

**Published:** 2025-05-08

**Authors:** Samantha N. Friday, Liezel A. Koellner, Spencer W. Brown, Christopher N. Calbat, Catherine F. Curran, Jordan D. Dietz, Andreas Koenig‐Dummer, Paul D. Friday, James A. Parker, Noah A. Simmons, Finnean A. Urmey, Alexis N. West, Sebastian G. Zagler, Ronald E. Viola, Christopher J. Halkides

**Affiliations:** ^1^ Department of Chemistry and Biochemistry University of Toledo Toledo Ohio USA; ^2^ Department of Chemistry and Biochemistry University of North Carolina Wilmington Wilmington North Carolina USA

**Keywords:** antifungal agents, covalent inactivators, Michael acceptors, vinyl sulfones

## Abstract

Aspartate semialdehyde dehydrogenase (ASADH) catalyzes the second step in the fungal pathway towards the synthesis of threonine, isoleucine, and methionine, and it has been identified as a viable target for antifungal drug development. Our previous work produced a group of vinyl sulfones that function as irreversible covalent inactivators of this enzyme. We have now expanded this initial set to produce vinyl sulfones with higher kinetic efficiency as covalent inactivators of ASADH purified from the pathogenic fungal species *Candida albicans*. The catalytic efficiency of these inactivators has also been compared to related classes of irreversible inactivators, vinyl sulfonamides, acrylamides, and sulfonyl ketones.

## 1 Introduction

Fungal infections are partially or wholly responsible for about 3 million deaths worldwide each year (Denning 2024). Unfortunately, the antifungal armentarium is severely limited, and the number of validated targets is quite small (Bouz and Doležal 2021). In addition, one of the leading antifungals, Amphotericin B, can produce severe kidney damage and even lead to death. Finally, the effectiveness of clinical antifungals is decreasing as resistant fungal organisms continue to emerge (Lee et al. 2023). In response to this worsening situation, the World Health Organization released a report (World Health Organization 2022) identifying *Candida albicans* and *Candida auris* as pathogens of critical priority, and several other candida species, including *C. parapsilosis, C. tropicalis, and C. glabrata*, as of high priority. This report recommends that research and development be directed toward the development of innovative antifungal agents (i.e., with no cross‐resistance to other antimicrobial classes, new chemical classes, new targets, and new modes of action with no or minimal drug‐drug interaction) that are effective against these priority pathogens.

**SCHEME 1:**
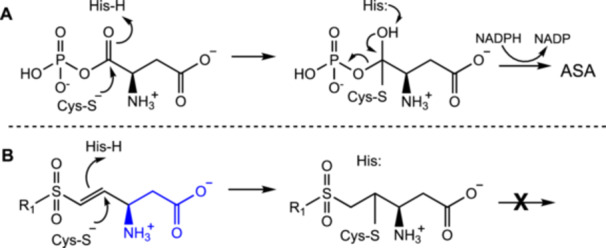
(A) Reaction mechanism for the ASADH‐catalyzed conversion of aspartyl phosphate to aspartate semialdehyde. (B) Proposed mechanism for the covalent inactivation of ASADH by vinyl sulfones.

Aspartate β‐semialdehyde dehydrogenase (ASADH) presents a potential drug target that can meet these criteria. ASADH catalyzes the reductive elimination of phosphate from aspartyl phosphate in the second step in the essential and uniquely microbial pathway to threonine, isoleucine, and methionine (Scheme 1). Binding of the anionic substrate is facilitated through salt bridge formation with two critical conserved arginine residues (Viola et al. 2011). Studies on the mechanism of the bacterial forms of ASADH demonstrate that a critical cysteine residue attacks the carbonyl carbon of the substrate, assisted by an active site histidine residue (Blanco et al. 2003, 2004b). Later studies on the fungal forms of ASADH indicate that, despite substantial differences in sequences, the active site residues are fully conserved among between the fungal and bacterial enzyme forms (Dahal and Viola 2018b).

Because fungal cells are eukaryotic, achieving selective toxicity against fungal species is significantly more difficult than achieving it against bacterial species. ASADH is essential to the survival of many microorganisms, including *C. albicans* (Dahal et al. 2020; Fu et al. 2021; Hill et al. 2015). Only a few antifungal agents have been directed against the aspartate pathway; for example, rhizocticin A inhibits threonine synthase, a downstream enzyme in this pathway (Kuplińska and Rząd 2021). However, no commercial antimicrobial agents have been produced that target ASADH. Since ASADH and the overall aspartate pathway are not found in mammals, the chances of achieving selectivity once antifungal drugs targeting this enzyme are produced is significantly enhanced.

The promise of developing irreversible inactivators into potent covalent drugs was fueled by their long residence times when bound to targets of interest. However, this initial promise of their unique mode of action was tempered by their high inherent reactivities, and by potential off‐target effects due to their perceived lack of specificity. These perceptions have slowed the development of selective covalent drugs, despite the fact that about 30% of the drugs that target enzymes, including aspirin and penicillin, were subsequently found to function by covalently modifying their target enzyme (De Cesco et al. 2017). However, over the past decade, the interest in covalent drug candidates has been rekindled (Singh 2022), as new synthetic approaches and new classes of inactivators have emerged, with the capability to fine‐tune their reactivity and focus their target selectivity. In addition, many of these new drug candidates have demonstrated a reduced risk of the development of resistance that can severely limit the lifetime of drug effectiveness (Bauer 2015).

Our previous work indicated that vinyl sulfones bearing aromatic rings at the β‐position can function as irreversible inhibitors of *Cal*ASADH (Friday et al. 2021). Our proposed mechanism for this inactivation is that the nucleophilic cysteine residue attacks the β‐carbon of the vinyl group and the conserved histidine active site residue protonates the α‐carbon of this covalent adduct to prevent reversal of the addition (Scheme 1). Docking studies also implicated a role for an additional arginine residue, Arg18, participating in binding with *para* substituents on the aromatic ring.

Here we report the synthesis and testing of β‐aromatic and β‐alanyl vinyl sulfone inhibitors that show significantly enhanced kinetic efficacy against our target enzyme compared to our previous β‐aromatic compounds. We have also compared the relative efficiency of one new vinyl sulfone to a structurally related vinyl sulfonamide and to a structurally related acrylamide. In contrast to these Michael acceptors, sulfonyl ketones have seldom been used in enzyme inhibition studies. All of these warheads except sulfonyl ketones are Michael acceptors, each of which are proposed to inactivate *Cal*ASADH by the same mechanism.

## 2 Results

### 2.1 Synthesis of Selective Enzyme Inactivators

The vast majority of enzyme inactivators incorporate a Michael acceptor into their structure to facilitate attack by an enzyme nucleophile. Reaction selectivity can then be achieved both through the fine‐tuning of the acceptor reactivity and through the addition of functional groups that enhance binding by mimicking the native substrates of a target enzyme. New compounds in several classes of potential inactivators have been synthesized and kinetically characterized against *Cal*ASADH to expand and develop this approach against an essential enzyme in fungal metabolism.

#### 2.1.1 Synthesis of β‐Aromatic Michael Acceptors

**SCHEME 2:**
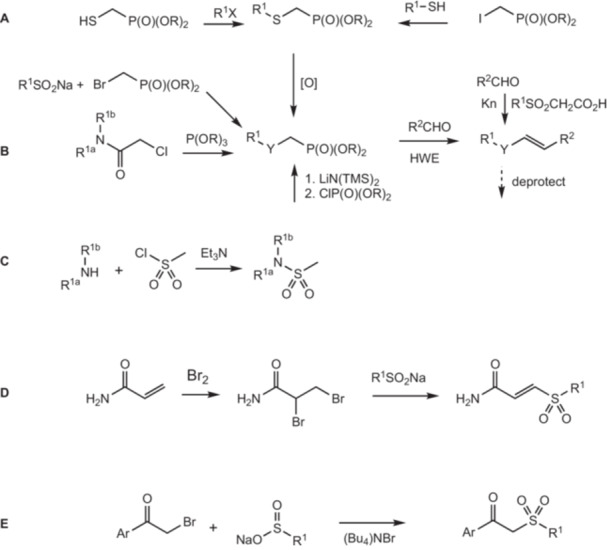
Synthetic routes for the production of different classes of inactivators. R = Et, iPr; R^1^ is one group when bound to S (Routes A, D, E) and two groups when bound to N (Routes B and C); R^2^ is alkyl or aryl. Multiple approaches were used for the synthesis of vinyl sulfones (A, Y is SO_2_ and R^1^ is alkyl or aryl), acrylamides (B, Y is C(O)N and R^1^ is alkyl or H), and vinyl sulfonamides (C, Y is SO_2_N and R^1^ is alkyl). Aldehydes were converted into alkenes using the Horner Wadsworth Emmons reaction (**HWE**) or the Knoevenagel reaction (**Kn**). When required, the resulting products were deprotected to yield the final compounds. Addition of Br_2_ to acrylamide (D, R^1^ is alkyl or aryl) followed by alkylation of sodium sulfinate anions produced sulfonyl acrylamides. Alkylation of sodium sulfonates with haloketones (E, R^1^ is aryl) produced sulfonyl ketones.

We have previously shown that aromatic vinyl sulfones are very good inactivators of ASADH, likely through covalent modification of the essential cysteine nucleophile within the active site of ASADH. As shown in Scheme 2, most of the compounds in this class were synthesized by the Masamune‐Roush variation (Ashburn et al. 2008) of the Horner Wadsworth Emmons (HWE) reaction. The phosphonate diester precursors utilized for the HWE reaction were produced by a variety of reactions. Sulfonyl phosphonates were made via three different tactics (Scheme 2, routes A‐B). One, diethyl thiomethylphosphonate (Choi et al. 2014) was alkylated with a haloalkane (Johnson et al. 2022), and the thioether was oxidized to the sulfone (Blumenkopf 1986). Two, a thiol was alkylated with an electrophilic phosphonate diester, and the thioether was oxidized (Evans et al. 2006). Three, a sodium sulfinate was coupled to an electrophilic phosphonate diester (Lizarzaburu et al. 2022). Unlike the reaction with sodium cyclopropylsulfinate, sodium 2‐propylsulfinate failed to produce any sulfonyl phosphonate, suggesting that this method is limited in scope. Availability or volatility of the thiol sometimes limits the scope of using XCH_2_P(O)(OR)_2_. Under those circumstances attack of HSCH_2_P(O)(OR)_2_ on an alkyl halide would be the method of choice for synthesizing vinyl sulfones. Carboxamidomethylphosphonate diesters were created through the Michaelis‐Arbuzov reaction (Ando et al. 2005). Sulfonylation of amines (Scheme 2, route C) produced sulfonamides (Zhou et al. 2008). Sulfonamides were treated with *n*‐butyllithium (El Hadri et al. 1995), or with lithium hexamethyldisilazane (Granberg et al. 2019), and then phosphorylated into the corresponding sulfamidomethylphosphonate diesters.

A dihydroxyformylbenzene was protected as its diacetyl derivative (Salomé et al. 2014), converted into a vinyl sulfone via the HWE reaction, and deprotected with hydrazine to regenerate the hydroxyl groups (Lang et al. 2008). One vinyl sulfone was synthesized via the Knoevenagel reaction (Friday et al. 2021) of an aldehyde with methylsulfonylacetic acid.

#### 2.1.2 Synthesis of β‐Alanyl Michael Acceptors

Amino acid‐bearing Michael acceptors were synthesized to more closely mimic the aspartyl phosphate substrate. A protected aspartic acid derivative, Boc‐Asp(OH)‐OtBu, was coupled to the Weinreb amine using *O*‐(Benzotriazol‐1‐yl)‐*N,N,N′,N′*‐tetramethyluronium hexafluorophosphate (HBTU) and triethylamine (Lee et al. 2009). This Weinreb amide was reduced to its aldehyde using diisobutylaluminum hydride (Zhang et al. 2015). Boc‐Asp(H)‐OtBu was converted into various vinyl derivatives using dialkyl phosphonate esters via the HWE reaction. The *tert*‐butylester and Boc protecting groups were removed with trifluoroacetic acid (Henderson and Phillips 2008), and the amino acids were purified and converted into hydrochloride salts with Dowex‐50.

#### 2.1.3 Synthesis of Sulfonyl Acrylamides

Based on inherent reactivity measurements, sulfonyl acrylamides were anticipated to be more active Michael acceptors than either vinyl sulfones or acrylamides. As shown in Scheme 2, route D, bromine was added to acrylamide, and the intermediate vicinal dibromide was reacted with an alkyl or aryl sodium sulfinate to produce sulfonyl acrylamides (Guan et al. 2007).

#### 2.1.4 Synthesis of Sulfonyl Ketones

Sulfonyl ketones are of interest as potential inactivators because they more closely mimic the mixed anhydride within the aspartyl phosphate substrate structure. Several sulfonyl ketones were purchased, and an additional compound in this class of inactivators was produced by alkylation of sodium methylsulfinate with a bromomethylketone (Scheme 2, route E) (Sunitha et al. 2008).

### 2.2 Docking Studies of Covalent Inactivators

**FIGURE 1:**
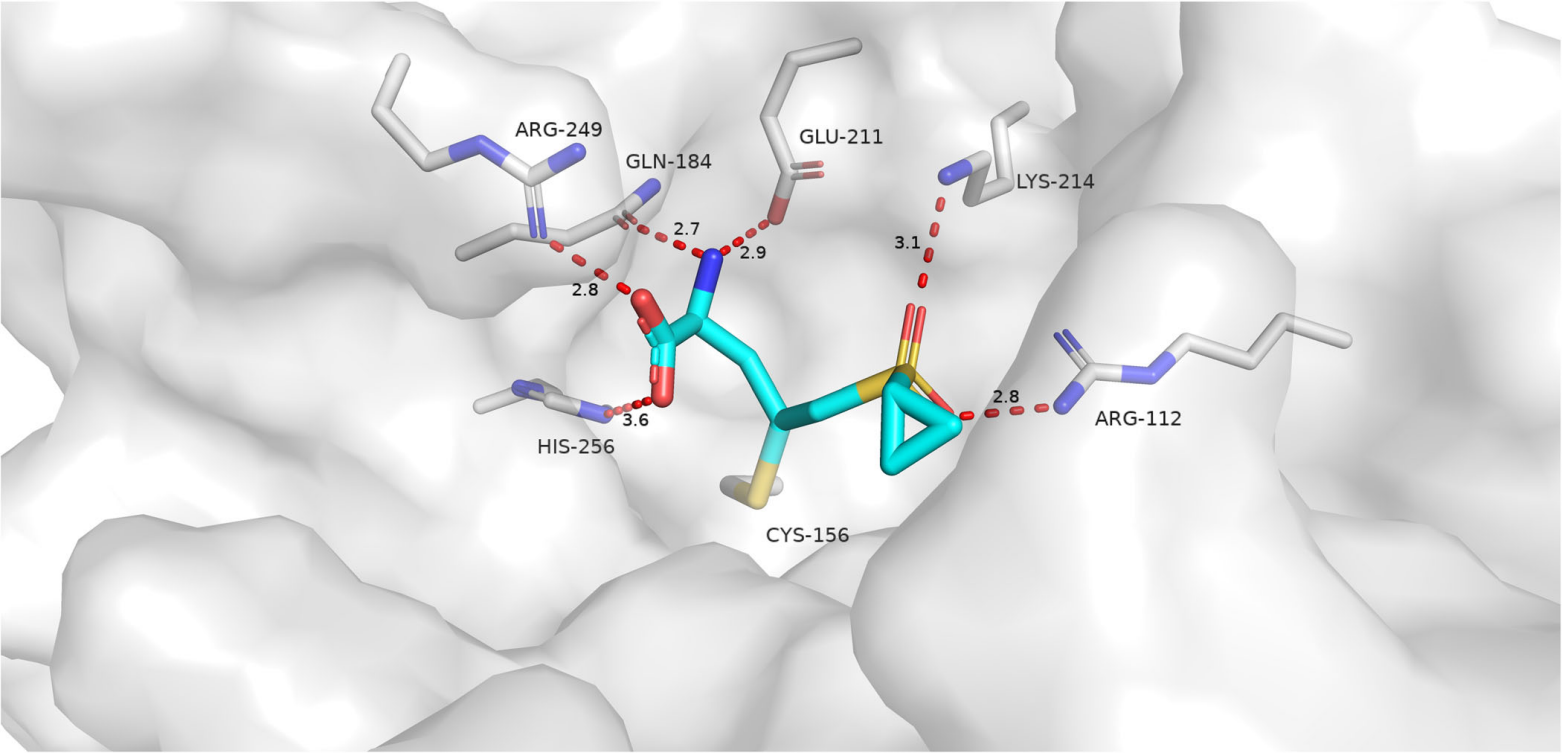
Cmpd 18 covalently docked to the catalytic cysteine (Cys‐156) in the active site of *Cal*ASADH. This compound is bound with the sulfone occupying the phosphoryl position near Arg‐112 and the amino acid positioned near Arg‐249 which is expected to bind the carboxylic acid of the substrate or product.

**FIGURE 2:**
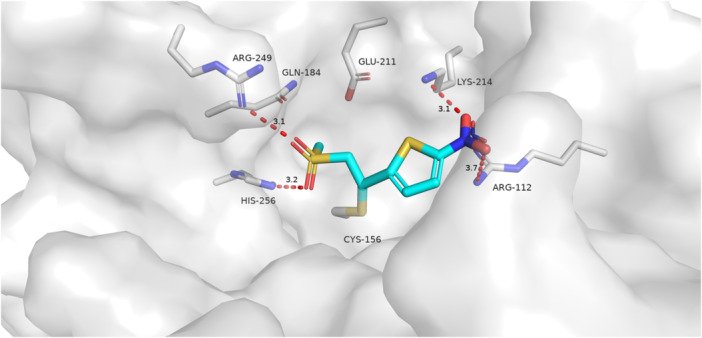
Cmpd 5 covalently docked to the catalytic cysteine (Cys‐156) in the active site of *Cal*ASADH. This compound is bound with the sulfone interacting Arg‐249, which is expected to bind the carboxylic acid of the substrate or product. The nitro group interacts with Lys‐214 and Arg‐112, groups that are involved in stabilizing the phosphoryl group of the substrate (Blanco et al. 2004a).

The structure of a fungal ASADH covalently inactivated has not yet been published. To aid in the design, synthesis, and optimization of selective inactivators, several compounds have been docked into the active site of *Cal*ASADH. Figure 1 shows a covalent docking pose of compound (cpd. 18) with a model of *Cal*ASADH. In this pose the sulfonyl group interacts with R112, in a similar manner to the phosphoryl group of the substrate analog aspartyl β‐difluorophosphonate (β‐AFP) in the structure of the *S. pneumoniae* ASADH•NADP complex (Pavlovsky et al. 2012). The carboxylate group of cpd. 18 interacts with R249, a result which is consistent with the somewhat disordered carboxylate group of β‐AFP. Figure 2 shows a covalent docking pose of cpd. 5 into the same structural model. In this pose the nitro group of cpd. 5 interacts with R112, and the sulfone interacts with R249, the reverse of the orientation that was observed for cpd. 18. This difference in orientation may reflect the greater size and rigidity of the nitrothiophene ring in cpd. 5 versus the amino acid chain in cpd. 18. Alternatively, an interaction of the α‐amino group of cpd. 18 with Glu‐211 may drive this change in orientation.

### 2.3 Irreversible Inactivation of Our Target Enzyme

With a set of newly synthesized compounds from different chemical classes now in hand, we next examined the kinetics of their interactions against ASADH purified from *C. albicans*. Our goal for these studies was to determine the relative efficiency of these compounds as potentially selective enzyme inactivators.

#### 2.3.1 Trends Among Aromatic or Heteroaromatic Vinyl Sulfones

Previously examined aromatic vinyl sulfones showed up to a 50‐fold change in inactivator efficiency against *Cal*ASADH as a result of changes in the aromatic group at R^2^ (Friday et al. 2021). Almost all of the variation among this initial set of compounds came from changes in their binding affinities (K_i_ values), with only a threefold variation in the rates of inactivation (*k*_inact_). As the nature of the aromatic group was expanded with newly synthesized compounds, the same trend was observed. The inactivation efficiencies of these newly synthesized set of aromatic vinyl sulfones, with a methyl group retained at the R^1^ position (Scheme 2), vary by over 1400‐fold, from the most efficient (Table 1, cpd. 8) to the least (cpd. 3). Again, most of this variation came from differences in K_i_ values, with only a fourfold maximum change in *k*_inact_.

**TABLE 1:** Vinyl sulfones and vinyl sulfonamides as inactivators against *C. albicans* ASADH.

	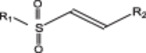				
Cpd no.	R_1_	R_2_	K_i_ (µM)	*k*_inact_ (min^‐1^)	*k*_inact_/K_i_ (M^‐1^ s^‐1^)
1	methyl	1,4‐phenol	538 ± 300	0.78 ± 0.34	20 ± 20
2	methyl	acetanilide	203 ± 83	0.39 ± 0.08	30 ± 10
3	methyl	benzamide	531 ± 134	0.42 ± 0.07	13 ± 4
4	methyl	5‐hydroxy‐2‐pyridine	0.61 ± 0.091	0.26 ± 0.014	7100 ± 1100
5	methyl	5‐nitrothiophene	2.10 ± 0.60	1.02 ± 0.10	8100 ± 2400
6	methyl	4‐quinoline	4.40 ± 0.89	0.95 ± 0.11	3600 ± 840
7	methyl	2‐quinoline	0.96 ± 0.24	0.67 ± 0.076	11,600 ± 3200
8	methyl	5‐isoquinoline	0.65 ± 0.11	0.73 ± 0.039	18,700 ± 3300
9	cyclopropyl	5‐isoquinoline	2.48 ± 0.41	0.78 ± 0.056	5200 ± 940
10	2‐pyridinyl	4‐pyridinyl	2.20 ± 0.18	0.42 ± 0.012	3200 ± 300
11	2‐morpholino	4‐pyridinyl	1.17 ± 0.13	0.26 ± 0.011	3700 ± 440
12	methyl	amide	2.70 ± 0.62	0.33 ± 0.020	2040 ± 480
13	phenyl	amide	2.30 ± 0.32	1.24 ± 0.077	8990 ± 1400
14	methyl	alanyl	7.6 ± 1.2	0.25 ± 0.015	550 ± 90
15	dimethylamino	alanyl	27.9 ± 4.3	3.20 ± 0.41	1910 ± 380
16	benzyl	alanyl	1.8 ± 0.82	0.27 ± 0.036	3000 ± 1000
17	isopropyl	alanyl	0.76 ± 0.15	0.26 ± 0.022	6000 ± 1000
18	cyclopropyl	alanyl	0.39 ± 0.093	0.31 ± 0.024	13,000 ± 3000

#### 2.3.2 Comparison of Benzene‐Derived Vinyl Sulfones

The inactivation efficiencies produced by cpds. 1‐3 are considerably slower than the rate observed for the previously examined 4‐benzoate and 4‐nitro derivatives (Friday et al. 2021). These latter compounds were the two fastest inhibitors previously characterized, and docking studies suggested that the polar/charged functional groups in the *para*‐position interact with Arg18 (Friday et al. 2021). The present data suggest that a hydrogen‐bond donor or a substituent larger than three atoms is disfavored in the *para* position (Scheme 4). The previously synthesized 3,4‐catechol derivative is a 100‐fold more efficient inhibitor than cpd. 1, suggesting that the hydrogen bonding patterns to ASADH are different between these two isomers.

**SCHEME 3:**
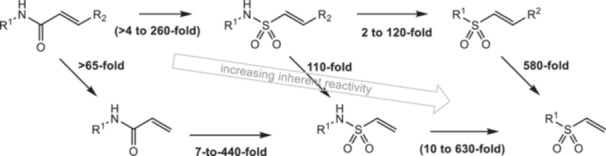
Summary of previous studies evaluating the nonenzymatic reactivities of acrylamides, vinyl sulfonamides, and vinyl sulfones (Hillebrand et al. 2024). Arrows show the increase in reactivity for pairs of molecules examined under identical conditions, with the enhanced reactivity in parentheses calculated from the related rate enhancements. The value of 580‐fold (Ábrányi ‐Balogh et al. 2018) refers to glutathione (GSH) in phosphate buffered saline (PBS). The value of 440‐fold (Kathman et al. 2014) refers to Ac‐Cys‐OMe in 2:1 PBS:DMSO at pD 8.0. The values of twofold and 110‐fold (Gehringer and Laufer 2019) refer to GSH in phosphate buffer at pH 7.4. The values of sevenfold and 65‐fold (Flanagan et al. 2014) refer to GSH in phosphate at pH 7.4. A value of ninefold for terminal vinyl sulfone versus acrylamide (Martin et al. 2019) refers to Boc‐Cys‐OMe in PBS. The value of 120‐fold (Reddick et al. 2003) refers to (phenylethyl)thiol in methanol with triethylamine.

#### 2.3.3 Comparison of Quinoline‐Containing Vinyl Sulfones

Relative to a previously synthesized inhibitor, (E)‐4‐(2‐methylsulfonyl)vinylpyridine, the newly synthesized cpd. 6, where the 4‐pyridinyl group is replaced by 4‐quinoline, is fivefold more efficient as judged by the second order rate constant, *k*_inact_/K_i_. Similarly, replacing the 2‐pyridinyl group in the previously synthesized inhibitor (E)‐2‐(2‐(methylsulfonyl)vinyl)pyridine with a 2‐quinolinyl group (Table 1, cpd. 7) evinces a twofold improvement in *k*_inact_/K_i_. Cpd. 8 is a reactive version of the known noncovalent inhibitor 5‐aminoisoquinoline (Dahal and Viola 2018a), and is found to be the fastest vinyl sulfones examined to date (Table 1). Replacement of the methyl group on sulfur with a cyclopropyl group (cpd. 9) unexpectedly reduced *k*_inact_/K_i_ by about threefold, opposite to the enhanced effect of the same substitution within the amino acid series (Section 2.4).

#### 2.3.4 Comparison of Pyridine‐ and Thiophene‐Derived Vinyl Sulfones

Cpd. 4 produces a twofold improvement in the value of *k*_inact_/K_i_ (5600 vs. 3200) over its nonhydroxylated version, (E)‐2‐(2‐(methylsulfonyl)vinyl)pyridine. Cpd. 7 is 1.6‐fold more reactive than cpd. 4, indicating a modest benefit of the second aromatic ring over the hydroxyl group on pyridine. Cpd. 10 is also 1.6‐fold faster than the previously characterized (E)‐4‐(2‐phenylsulfonyl)vinylpyridine; where the 2‐pyridyl group in cpd. 10 is isosteric to the phenyl group. Cpd. 5 is about twofold faster than the previously characterized (E)‐2‐(methylsulfonyl)vinyl‐4‐nitrobenzene.

### 2.4 Comparison of Amino Acid Vinyl Sulfones

A set of amino acid‐containing vinyl sulfones were synthesized with the aim of producing inactivators that more closely match the structural properties of the ASADH substrate. The sulfonyl group was hypothesized to mimic the phosphoryl group of aspartyl phosphate, while the amino acid moiety matches the substrate aspartyl group. A methyl group was initially hypothesized to be the best choice for the third group on the sulfonyl moiety, being closest in size to the phosphoryl oxygen of the substrate. On the basis of the second order rate constant, *k*_inact_/K_i_ values, the observed order of inactivator efficiency is cyclopropyl > isopropyl > benzyl > methyl, with more than a 20‐fold change from the most to least active (Table 1). For three of the groups, smaller size correlates with more efficient inactivation, but the methyl group deviates from this trend.

### 2.5 Comparison of Sulfonyl Ketones

Sulfonyl ketones are among the least studied class of potential enzyme inactivators. The simplest methylsulfonyl ketone (Table 2, cpd. 19) showed the least activity, suggesting that the presence of an aromatic ring adjacent to the carbonyl group is needed to improve activity. Among the series of aromatic methylsulfonyl ketones studied, the order of inactivator efficiency was: phenyl > 2‐pyridinyl > 3‐pyridinyl > 4‐pyridinyl (Table 2). This order is different from the previously studied vinyl sulfones with the same substituents at R_2_, where the 2‐pyridinyl derivative was the most efficient (Friday et al. 2021). These differences suggest that the change in warhead affects which aromatic ring provides the greatest activity.

**TABLE 2:** Sulfonyl ketones as inactivators against *C. albicans* ASADH.

	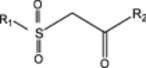				
Cpd. no.	R_1_	R_2_	K_i_ (µM)	*k*_inact_ (min^‐1^)	*k*_inact_/K_i_ (M^‐1^ s^‐1^)
19	methyl	methyl	31.7 ± 4.9	0.30 ± 0.019	160 ± 26
20	methyl	4‐pyridinyl	59 ± 22	1.11 ± 0.22	310 ± 130
21	methyl	3‐pyridinyl	25.9 ± 6.6	0.72 ± 0.10	460 ± 130
22	methyl	2‐pyridinyl	6.08 ± 0.76	0.30 ± 0.020	820 ± 115
23	methyl	phenyl	2.2 ± 0.77	0.31 ± 0.037	2,350 ± 870

### 2.6 Comparison of Vinyl Sulfone, Vinyl Sulfonamide and Acrylamide Efficiencies

The primary issues that must be addressed to inactivate a specific target are the inherent compound reactivity and target selectivity. Several acrylamides were synthesized to examine a direct comparison with vinyl sulfone reactivity against *Cal*ASADH. Comparison of a morpholino/pyridinyl vinyl sulfonamide with moderate inactivator efficiency (Table 1, cpd. 11) to the corresponding acrylamide (Table 3, cpd. 24) found that the acrylamide is a threefold less efficient inactivator of *Cal*ASADH. Changing the R^1^ group from 2‐morpholino to amino produced an acrylamide that should more closely resemble the substrate structure in size, yet no change was observed in the respective kinetic values (Table 3, cpd. 25).

**TABLE 3:** Acrylamides as inactivators against *C. albicans* ASADH.

	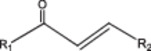				
Cpd. no.	R_1_	R_2_	K_i_ (µM)	*k*_inact_ (min^‐1^)	*k*_inact_/K_i_ (M^‐1^ s^‐1^)
24	morpholino	4‐pyridinyl	4.41 ± 0.85	0.29 ± 0.025	1100 ± 200
25	amino	4‐pyridinyl	3.79 ± 1.60	0.26 ± 0.050	1140 ± 530
26	amino	alanyl	1.46 ± 0.27	0.69 ± 0.050	7900 ± 1600
27	dimethylamino	alanyl	1.43 ± 0.18	0.72 ± 0.035	8400 ± 1100

A comparison between an amino acid‐containing vinyl sulfone and the corresponding acrylamide produced a different result versus the same comparison when the β‐substituent was the 4‐pyridyl group. A dimethylamino/alanyl acrylamide (Table 3, cpd. 27) was found to be slightly more efficient than the corresponding vinyl sulfone (Table 1, cpd. 15). Once again, replacing the dimethylamino group at R^1^ of the acrylamide with an amino group (Table 3, cpd. 26) had no effect on the kinetic values.

### 2.7 In Vitro Antimicrobial Activity

The next question to be addressed is whether the most efficient inactivators against the purified *Cal*ASADH will show corresponding activity against the growth of a *C. albicans* cell line. Compounds were initially screened using disk‐diffusion assays on agar plates (Table S1), and those showing a zone of inhibition were tested in liquid culture. Not unexpectedly, most of the ASADH inactivators did not show appreciable inhibition when examined in this disk diffusion assay. When tested against growing cells, cpds. 5‐7 were weakly inhibitory against *C. albicans* in liquid culture (Table 4); cpds. 5 and 7 are moderately active against ASADH. Thus, there is some correlation between the rate constants for enzyme inactivation and the activity against microorganisms in vitro. Cpd. 6 and (E)‐(2‐trifluoromethylsulfonyl)vinylbenzene are less active against ASADH but still give some inhibition against *C. albicans* growth. However, cpd. 8 is among the most potent inactivators against ASADH, yet it did not demonstrate any activity against whole *C. albicans* cells. Quinolines were previously identified as inhibitors of ASADH and other enzymes in the aspartate pathway (Musiol et al. 2010). While ASADH is the most likely primary target, we recognize the possibility that another enzyme in addition to ASADH could also be a target in these whole cell assays.

**TABLE 4:** Inhibition of *C. albicans* growth^a^.

Inactivator	*MIC, µg/mL*
16	> 500
7	250
(E)‐2‐(Trifluoromethylsulfonyl)vinylbenzene	125
5	125
6	250

^a^ Minimum inhibitory concentration (MIC) determined from three independent experiments.

## 3 Discussion

### 3.1 Comparison of Warhead Inherent Reactivities

In addition to the classical properties that are typically used to evaluate compounds as effective drug candidates (Lipinski et al. 2001), compounds containing a covalent warhead must be capable of selectively reacting with its target nucleophile while minimizing off‐target interactions. The two properties that influence target selectivity are the affinity of a compound for binding to the target (K_i_) and the inherent reactivity of the compound once bound (*k*_inact_), with this ratio (*k*_inact_/K_i_) serving as a measure of inactivator efficiency. Incorporating a tunable functional group into a compound designed to resemble the natural substrate of a target enzyme will help to maximize this efficiency.

The vinyl sulfones, vinyl sulfonamides, and acrylamides examined in this study are all Michael acceptors, a major class of covalent warheads. When examined in nonenzymatic thiol‐Michael addition reactions (Scheme 3), disubstituted vinyl sulfonamides were found to be between twofold and 120‐fold less reactive than disubstituted vinyl sulfones (Gehringer and Laufer 2019; Reddick et al. 2003). This decrease in reactivity is qualitatively similar to the decrease in reactivity from vinyl ketones to acrylamides. Terminal acrylamides are sevenfold to 440‐fold less reactive than terminal vinyl sulfonamides (Flanagan et al. 2014; Kathman et al. 2014). Therefore, reactivity increases in the order acrylamide to vinyl sulfonamde to vinyl sulfone.

**SCHEME 4:**
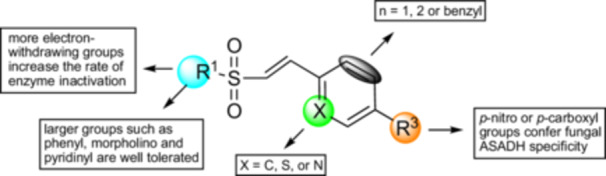
Structure‐activity relationships among aryl vinyl sulfones. Electron‐withdrawing groups on sulfur (R^1^) increase rate of inactivation. Aromatic and heteroaromatic rings (R^2^ in Scheme 2) are well tolerated at the β‐carbon, as is the alanyl group. Quinolines and pyridines with multiple regiochemical orientations of the nitrogen versus the vinyl group. H‐bond acceptors (R^3^) *para* with respect to the vinyl group improve binding.

Internal vinyl sulfonamides are 110‐fold less reactive than terminal ones; internal vinyl sulfones are 580‐fold less reactive than terminal ones (Ábrányi‐Balogh et al. 2018; Gehringer and Laufer 2019). An increase in the rate of thiol‐Michael addition of at least 65‐fold is also correlated with a lower degree of substitution of acrylamides, qualitatively similar to the effect of decreased substitution on the rate of addition to vinyl sulfones (Flanagan et al. 2014). Therefore, reactivity increases with decreasing substitution of the double bond (Scheme 3). Moreover, previous studies have indicated that there are other avenues to tune the nonenzymatic reactivity of Michael acceptors. For example, attaching electron withdrawing groups to a sulfonyl moiety increased the inherent reactivity of vinyl sulfones (Kiemele et al. 2016).

In addition to changes in inherent reactivity, two structural features may also enhance the selectivity for ASADH. First, the reactive functional group in vinyl sulfones and vinyl sulfonamides have a tetrahedral sulfur atom with two oxygen atoms that can accept hydrogen bonds, thereby mimicking the properties of a phosphoryl group (Wong et al. 2009). In contrast to this excellent structural match of the sulfonyl group, the carbonyl group within acrylamides is smaller, and the carbon atom is planar; therefore, it is less overtly similar to the phosphoryl group. The P‐O‐C bond angle in methyl phosphate is 121°, similar to typical C = C‐Y angles in vinyl derivatives (Klooster and Craven 1992). The β‐carbon of a vinyl sulfone is sp^2^‐hybridized, as is C‐4 of the substrate, aspartyl phosphate (Scheme 1). These geometric and chemical properties show that vinyl sulfones and vinyl sulfonamides are isosteric to the mixed anhydride moiety within aspartyl phosphate. This suggestion is similar to the recent observation that vinyl sulfones can also mimic vinyl ketones (Xiao and Chen 2024). Second, vinyl sulfonamides and acrylamides have a nitrogen atom that can accept a hydrogen bond, similar to the properties of a phosphoryl oxygen atom. The effects of substituents on these structural features can then be used to fine tune enzymatic reactivity and selectivity, even among different orthologs of ASADH.

### 3.2 Comparison of Warhead Reactivities Against ASADH

A series of three amino acids was synthesized to compare vinyl sulfones with vinyl sulfonamides and with acrylamides. In all three cases the substituents on the atom adjacent to the sulfonyl or carbonyl group were methyl groups. As judged by the second order rate constants, *k*_inact_/K_i_, the acrylamide (cpd. 27) was most efficient, followed closely by the vinyl sulfone (cpd. 17) and the vinyl sulfonamide (cpd. 15). Neither the second order nor the first order rate constants for inactivation, *k*_inact_, correspond with the relative nonenzymatic reactivities of the three electrophiles (Scheme 3), suggesting that the environment and reactivity of the enzyme nucleophile plays a significant role in the efficiency of ASADH inactivators. The vinyl sulfonamide (cpd. 11) is more efficient, however, than the corresponding acrylamide (cpd. 16), opposite to the order of compounds 27 versus 15. This difference indicates that the 4‐pyridyl substituent produces a different trend in warhead behavior from the alanyl substituent. The sulfonyl acrylamides, cpds. 12 and 13 each have two electron‐withdrawing groups on the alkene, and the S‐phenyl derivative (cpd. 13) shows elevated first‐order and second‐order rate constants for inactivation over the S‐methyl derivative (cpd. 12).

### 3.3 Effect of β‐Substituents on Inactivation Kinetics

We also synthesized a series of novel Michael acceptors bearing an alanyl group at the β‐position to complement new and previously synthesized aromatic β‐substituents. The best examples of both types of β‐substituents produced more efficient inactivation than previously synthesized vinyl sulfones (Friday et al. 2021). Despite the presence of the α‐amino group that more closely resembles the substrate structure, the kinetic results do not indicate that these amino acid derivatives have a greater affinity for ASADH than the aromatics. In two instances, previously identified excellent noncovalent inhibitors of a fungal ASADH (Dahal and Viola 2018b) or a bacterial ASADH (Wang et al. 2021) inspired the design of the structurally‐related covalent inactivators, cpds. 5 and 8. Both of these new compounds gave more rapid second‐order rate constants of inactivation than the other members of this class of vinyl sulfones.

We also varied the substituent at the sulfonyl or carbonyl group to expand our study on the effects of electron‐withdrawing or releasing groups, as judged by the Hammett parameter, σ_meta_. The 2‐pyridyl group (σ_m_ 0.33) produced a smaller increase in *k*_inact_ versus phenyl (σ_m_ 0.06) than the trifluoromethyl group (σ_m_ 0.43) produced versus the methyl group (σ_m_ ‐0.07). This result is qualitatively in accord with the greater difference in electron withdrawing abilities of the CF_3_ group over the CH_3_ group versus the 2‐pyridyl group over the phenyl group (Friday et al. 2021).

For the reasons already given, we hypothesized (*vide supra*) that the sulfonyl group would bind in a similar manner to the phosphoryl group. The choice of substituents on the sulfonyl or carbonyl group gave some unexpected results, both with respect to the presence or absence of hydrogen bond donors and to the nature of the alkyl or aryl group present. The presence of H‐bond donors at the portion of the active site binding the phosphoryl group would be expected to repel a H‐bond donating group on the inactivator, but an inhibitor bearing a ‐NH_2_ group (cpd. 26) was similar in reactivity to an inhibitor bearing a ‐N(CH_3_)_2_ group (cpd. 27). The cyclopropyl group is significantly larger and more lipophilic than a methyl group. This change results in improved binding when the β‐substituent is alanine (cpd. 14 vs. 18), but weakened when the β‐substituent is 5‐isoquinoline (cpd. 8 vs. 9). An explanation consistent with the docking studies is that the cyclopropyl group of each inhibitor binds in a different location of the active site.

### 3.4 In Vitro Activity of Covalent ASADH Inactivators

Antifungal activity against *C. albicans* depends both upon the ability of a compound to diffuse across the envelope and then to locate and inactivate the target enzyme. The fact that some vinyl sulfones can inhibit *C. albicans* growth (Table 4) is not unexpected, given their potent capability to inactivate ASADH. However, the amino acid derivatives such as cpd. 16 did not show significant in vitro inhibition against *C. albicans* (Table S1). Several factors could be responsible for this lack of inhibition. The group of amino acid containing vinyl sulfones are more charged and less hydrophobic than the other inactivators, decreasing their potential for passive diffusion. Also, the canonical amino acids present in the growth media will likely compete against the inhibitors for binding to amino acid transporters, as was observed in the case of oxalysine and L‐lysine (Basrai et al. 1992). More generally, the potency of drug candidates directed against enzymes within the aspartate pathway of *C. albicans* or other microorganisms may depend strongly on the choice of growth medium (Skwarecki et al. 2018). It is always possible that some nucleophiles present in the growth media could have reacted with some fraction of these compounds, thereby decreasing their overall apparent reactivity.

## 4 Conclusions and Prospects

We have synthesized a wider range of aromatic vinyl sulfones with improved kinetics of inactivation, and most of the aromatic vinyl sulfones are electrically neutral to allow passive diffusion into fungal cells. We also synthesized some novel amino acids vinyl sulfones, the best of which have kinetics of activation that are comparable to the best aromatic vinyl sulfones. In addition, the amino acid bearing vinyl groups can be more easily improved via structure activity relationship modifications than some of the known inhibitors of amino acid biosynthetic pathways. Compounds in all three classes, vinyl sulfones, vinyl sulfonamides, and acrylamides, are effective ASADH inactivators; therefore, all three classes have the potential for further development. The alkenes in this study are each internal, lowering their nonenzymatic reactivity relative to terminal alkenes and potentially improving their in vivo selectivity. Sulfonyl ketones sterically resemble mixed anhydrides, yet they have seldom been explored as enzyme inactivators (Iqbal et al. 2020). Future studies will be directed toward structural modifications of these classes of inactivators to improve their antimicrobial potency, and by conjugation with molecules that can be recognized by a transporter to confer improved fungal cellular uptake.

## 5 Experimental Section

### 5.1 General Synthetic Methods

Diethyl methylsulfonylmethylphosphonate (DMeSUMP) was synthesized as previously described (Friday et al. 2021). Triethylamine was stirred with calcium hydride and distilled under dry conditions. Reagents and dry solvents (DCM, DMF, and THF) were obtained commercially and used without purification. Glassware was oven‐dried, and reactions were performed under a nitrogen atmosphere, except for reactions using Oxone. Each reagent flask in Horner Wadsworth Emmons reactions was rinsed with one or two small portions of ACN. Silica columns were usually dry loaded. Thin‐layer chromatographic spots were detected using fluorescence quenching, phosphomolybdic acid (PMA), ceric ammonium molybdate, potassium permanganate, ninhydrin, or dinitrophenyl hydrazine. Residual trifluoroacetic acid was typically removed by stripping with toluene. Analytical grade Dowex 50 resin was rinsed with small portions of ethanol followed by water. NMR spectra were acquired on a Bruker Avance III 300 NMR spectrometer. Chemical shifts are reported in ppm, and coupling constants are reported in Hz. LC‐MS chromatograms and spectra were acquired using a gradient of acetonitrile in water on a Waters Arc HPLC coupled to an Acquity QDa mass spectrometer.

### 5.2 Purification of ASADH

*C. albicans* ASADH (*Cal*ASADH) was purified as previously described (Friday et al. 2021).

### 5.3 Evaluation of ASADH Inactivation

**FIGURE 3:**
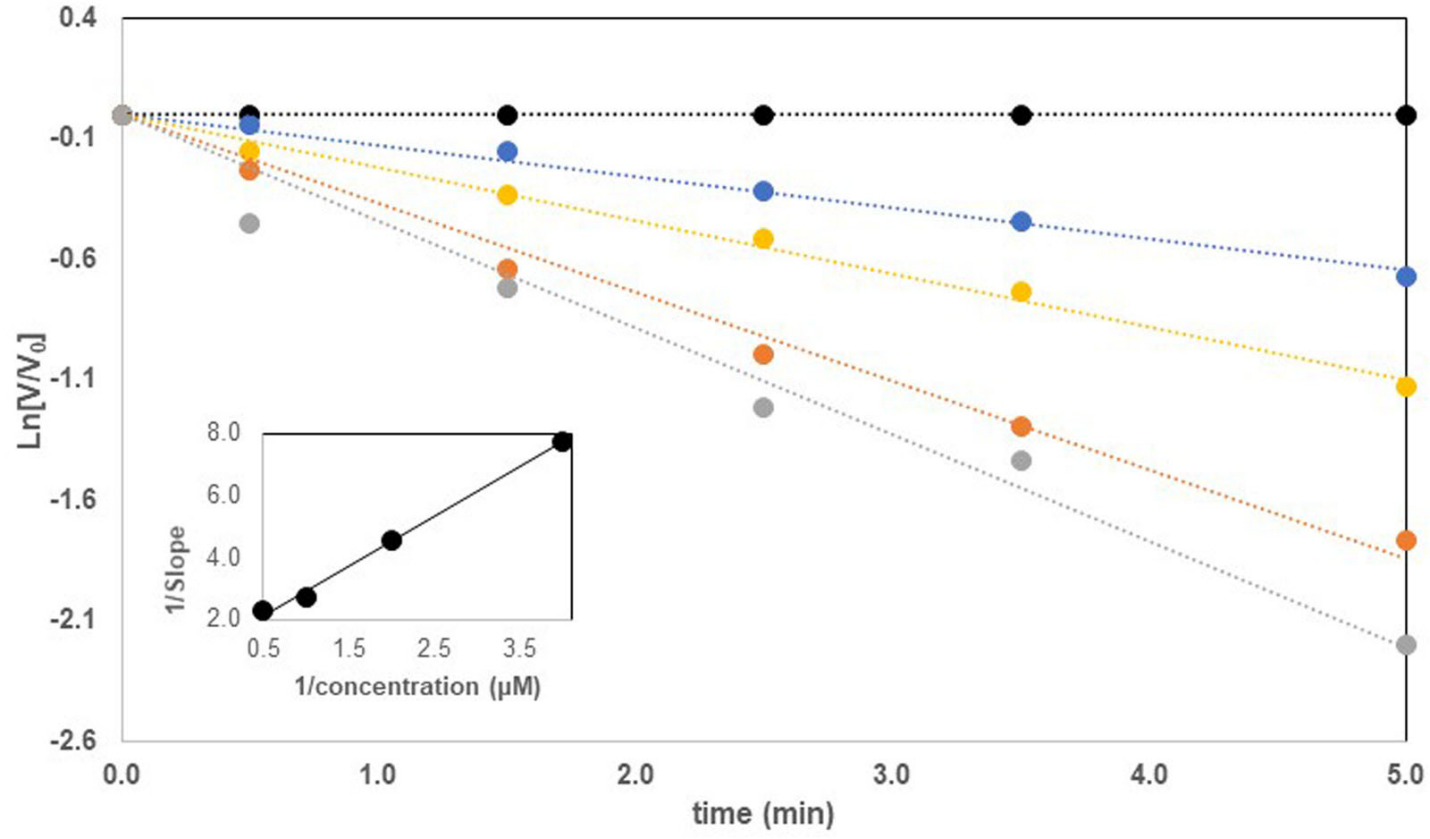
Representative inactivation of *C. albicans* ASADH by a vinyl sulfone. Plot of the natural log of the ratio of enzyme velocity in the presence of inactivator (v) to the control rate without inactivator (v_o_) versus time. Inactivator was added at t = 0, at concentrations of 0 (•), 0.25 (•), 0.5 (•), 1.0 (•) and 2.0 µM (•). Inset shows the reciprocal plot of inactivation rate versus inactivator concentration that is used for the preliminary estimate of K_i_ and *k*_inact_, followed by nonlinear fitting to determine the final values.

The substrate aspartate β‐semialdehyde was synthesized as previously described (Friday et al. 2021). The inactivation studies involved incubating varying concentrations of each potential inactivator with 50 µL of *Cal*ASADH (30 µg/mL) for each reaction. At selected time points a 10 µL aliquot was withdrawn and added to 190 µL of the assay mixture containing 120 mM CHES (pH 8.6), 120 mM KCl, 0.3 mM L‐aspartate‐β‐semialdehyde (ASA), 20 mM phosphate and 1.5 mM nicotinamide adenine dinucleotide phosphate (NADP). Production of reduced nicotinamide adenine dinucleotide phosphate was monitored at 340 nm in a SpectraMax 190 plate reader, with the tangent to the reaction time course used to determine the reaction rate at each time point. A plot of the natural log of the rate of the reaction divided by the control rate in the absence of inactivator (ln [v/v_0_]) versus time of incubation was used to calculate the observed rate constant of inactivation at each inactivator concentration (see Figure 3). A replot of the rate of inactivation versus inactivator concentration allows initial estimates of the *k*_inact_ (min^‐1^), the K_i_ value (µM), and the inactivator efficiency, *k*_inact_/K_i_ (M^‐1^s^‐1^). The best determination of these parameters was obtained via nonlinear regression using ProStat (Poly Software International) to fit the following equation:

$$k_{\mathrm{obs}}=k_{\mathrm{inact}}[I]/\left(K_{i}+\left[I\right]\right)$$

### 5.4 Docking Studies

The *Cal*ASADH crystal structure with PDB code 3hsk served as the model for molecular docking studies (Arachea et al. 2010). Cpd. 18 (in the S conformation) and cpd. 5 were chosen as ligands. Water molecules and NADP were removed before protein and ligand preparation through the Schrodinger Suite 2024‐1. Ligand poses were generated using Schrodinger Covalent Docking against the active‐site cysteine‐156 (Zhu et al. 2014). As a part of its covalent docking workflow, Schrodinger chose the lower energy configuration at the β‐carbon of the Michael acceptor for cmpd 5 (R) and cmpd 18 (S); these are the same relative configuration. The top 3 poses for each ligand were examined, and the most plausible binding conformations were displayed.

### 5.5 *C. albicans* Growth Inhibition Studies

A microbroth dilution assay was conducted to determine the minimum inhibitory concentration (MIC) of each test compound. The protocol for this assay was based on Clinical and Laboratory Standards Institute (CSLI) guidelines (CLSI Supplement M100, 2018). Compounds or control (nystatin, Alfa Aesar, Ward Hill MA) were dissolved in DMSO to 10 mg/mL. Each well of a 96‐well microtiter plate (200 µL total volume) contained 1% yeast extract‐2% peptone‐2% dextrose broth (YPDB), serial twofold dilutions of each compound (from 500 µg/mL to 0.5 µg/mL), 5% DMSO, and *Candida albicans* (ATCC 10231) at 5 × 10^5^ cfu/mL. Control wells contained YPDB, 5% DMSO, either with (positive) or without (negative) *C. albicans* cells. Following incubation at 30°C for 18–24 h, 50 µL of 0.2 mg/mL resazurin sodium salt (Thermo Fisher Scientific, Eugene OR) in YPDB was added to each well. The plate was incubated at 30°C for another 1–4 h for the color to develop. The MIC was taken as the last well with a purple color indicating no cell growth. Each MIC was calculated as the mean of triplicate assays.

## Author Contributions

S.N.F. carried out all of the kinetic evaluations of the enzyme inactivators, and L.A.K. was involved in the synthesis and characterization of multiple enzyme inactivators. N.A.S., J.D.D., C.F.C., J.A.P., A.K.‐D., C.N.C., S.W.B., and F.A.U. were each involved in the synthesis and characterization of several of the enzyme inactivators. P.D.F. purified the *Cal*ASADH used in the kinetic studies and assisted with the kinetic evaluations. S.G.Z. executed the docking studies and A.N.W. carried out the disk diffusion and growth inhibition studies. R.E.V. and C.J.H. designed, coordinated, and supervised the overall project, and wrote the final manuscript.

## Conflicts of Interest

The authors declare no conflicts of interest.

## Supporting information

Supporting Information v16 CJH.

## Data Availability

The data that support the findings of this study are available from the corresponding author upon reasonable request.
