# Supplementary material for: Development of Efficient Covalent Inactivators of a Fungal Aspartate Semialdehyde Dehydrogenase
Source: Drug Dev Res. 2025 May 8;86(3):e70095. doi: 10.1002/ddr.70095 (PMC12060211; doi:10.1002/ddr.70095)
Supplement: Supplementary file 1 — Supporting Information v16 CJH. [file DDR-86-e70095-s001.docx]

**Supporting Information**

**1. Compound Syntheses**

**Compound 1: (E)-1,4-Dihydroxy-2-(2-methylsulfonyl)vinylbenzene**

**1A** 2,5-Diacetoxybenzaldehyde

2,5-Dihydroxbenzaldehyde (1.500 g, 10.86 mmol) and K_2_CO_3_ (3.00 g, 21.7 mmol) in diethyl ether (50 mL) were stirred at room temperature, and acetic anhydride (5.41 g, 53.0 mmol) was added dropwise. The mixture was stirred for 3 hours and filtered. The organic solution was extracted with saturated Na_2_CO_3_ and brine, and the organic layer was dried with MgSO_4_. The volatiles removed with rotary evaporation and high vacuum. The crude product was triturated using pentane and filtered to generate a solid (0.438 g, 18.2% yield).

H-1 NMR

10.10 (s, 1H), 7.64 (d, J = 2.4, 1H), 7.37 (td, J = 8.7, 23.0, 1H), 7.30-7.22 (m, 1H), 2.41 (s, 3H), 2.34 (s, 3H).

C-13 NMR

195.75, 169.53, 169.07, 148.97, 148.46, 130.70, 124.56, 123.35, 118.72, 21.00, 20.81.

**1B** 1,4-Diacetoxy-2-(methylsulfonyl)vinylbenzene

Lithium chloride (0.276 g, 6.51 mmol), was suspended in ACN and stirred at room temperature. Successively were added DMeSUMP (0.982 g, 4.27 mmol), DBU (0.541 g, 3.56 mmol), and protected aldehyde 1A (0.790 g, 3.56 mmol) in a small portion of ACN; the last solution was added dropwise. The reaction was stirred overnight and quenched with saturated NH_4_Cl. After the volatiles were removed and a small portion of water added, the solution was extracted four times with DCM. The organic layer was dried with Na_2_SO_4_, filtered, and the solvent was removed with rotary evaporation. The crude product was eluted from silica using 50:50 to 70:30 ethyl acetate/hexanes. The volatiles were removed with rotary evaporation and high vacuum to provide the product (0.264 g) in 24.9% yield. A second, less pure pool (0.356 g) was obtained in 38% yield.

H-1 NMR

7.68 (d, J = 15.6, 1H), 7.37 (t, J = 1.4, 1H), 7.24-7.21 (m, 2H), 6.91 (d, J = 15.3, 1H), 3.01 (s, 3H), 2.38 (s, 3H), 2.32 (s, 3H).

C-13 NMR

169.07, 168.84, 148.32, 146.94, 136.78, 129.14, 125.81, 125.34, 124.38, 120.75, 43.13, 21.05, 20.96.

**1** (E)-1,4-Dihydroxy-2-(2-methylsulfonyl)vinylbenzene

The protected vinyl sulfone 1B (0.261 g, 0.875 mmol) was dissolved in ACN (7.2 mL) and stirred at room temperature. Hydrazine monohydrate (0.141 mL, 2.90 mmol) was added and the reaction stirred for 2 hrs. The reaction was quenched with saturated NH_4_Cl (8 mL) and the volatiles were removed by rotary evaporation. The product was dissolved in 0.5 M HCl (3.3 mL) to remove hydrazine and its acetylated derivative, and this solution was extracted with 3 8.5-mL portions of ethyl acetate. The combined organic layers were washed with brine (4 mL), dried with Na_2_SO_4_, and the solvent was removed with rotary evaporation. The crude product was combined with a 1.026-mmol portion from a second synthesis, and the compound was eluted from silica using 6% ethanol in DCM. The solvent was removed with rotary evaporation and high vacuum to obtain the product (0.225 g, 55.3% yield).

H-1 NMR (d_6_-acetone)

7.74, 7.26 (ABq, J = 15.6, 2H), 7.03 (d, J = 2.7, 1H), 6.89-6.80 (m, 2H), 3.03 (s, 3H).

C-13 NMR (d_6_-acetone)

150.49, 150.14, 137.96, 127.39, 119.73, 119.62, 117.01, 114.76, 42.42.

HRMS calc. for C_9_H_9_O_4_S (M-1) m/z 213.022157; found m/z 213.0223.

**Compound 2: (E)-4-(2-Methylsulfonyl)vinylacetanilide**

LiCl (0.095 g, 2.24 mmol) was suspended in ACN (14 mL) and stirred at room temperature. Successively were added ACN solutions of DMeSUMP (0.2813 g, 1.22 mmol), DBU (0.1916 g, 1.26 mmol), and 4-acetamidobenzaldehyde (0.2040 g, 1.25 mmol); the last reagent solution was added dropwise. The reaction was stirred overnight and was quenched with a 4-mL portion of saturated NH_4_Cl. The volatiles were removed with rotary evaporation. After a 5-mL portion of water was added, the aqueous layer was extracted with 4 portions of DCM, each of ~12 mL. After the combined organic layers were dried with MgSO_4_, the solvent was removed with rotary evaporation. The crude product was eluted from silica using 96:4 DCM/2-propanol. Removal of the solvent provided the solid (0.238 g, 82% yield).

H-1 NMR

7.78-7.64 (multiplet, 4H), 7.48 (m, J = 15.6), 7.19 (m, J = 15.6, 1H), 3.03 (s, 3H), 2.10 (s, 3H)

C-13 NMR (d_6_-acetone)

168.39, 142.05, 141.69, 129.45, 127.43, 126.05, 119.05, 42.40, 23.41.

HRMS calc. for C_11_H_14_NO_3_S (M+1) m/z 240.0694; found m/z 240.0672.

**Compound 3: (E)-4-(2-Methylsulfonyl)vinylbenzamide**

LiCl (0.270 g, 6.37 mmol) was suspended in ACN (33 mL) and stirred at room temperature. Successively were added DMeSUMP (0.928 g, 4.03), DBU (0.568 g, 3.73), and 4-formylbenzamide (0.485 g, 3.25 mmol) in DMF (4 mL); the last reagent was added dropwise. The reaction was quenched with saturated NH_4_Cl (11 mL), and the volatiles were removed with rotary evaporation. After a small portion of water was added, the solution was extracted with four 20-mL portions of DCM; the combined organic layers were dried with MgSO_4_; and the solvent was removed with rotary evaporation. The product was eluted from silica using EtOAc. The product was repurified over silica using 30:70 acetone/DCM. Removal of the solvent left a colorless product (0.006 g, 1% yield).

H-1 NMR (d_6_-acetone)

8.04-7.80 (m, 4H), 7.60 (m, J =15.6, 1H), 7.59 (broad s, 1H), 7.44 (m, J = 15.6, 1H), 6.79 (broad s, 1H), 3.08 (s, 3H).

C-13 NMR (d_6_-DMSO)

167.53, 140.73, 136.52, 135.52, 130.31, 129.03, 128.52, 43.05.

HRMS calc. for C_10_H_12_NO_3_S (M+1) m/z 226.0538; found m/z 226.0518.

**Compound 4: (E)-5-Hydroxy-2-(2-methylsulfonyl)vinylpyridine**

5-hydroxypyridine-2-carbaldehyde (0.0811 g, 0.659 mmol), methylsulfonylacetic acid (0.1826 g, 1.32 mmol), pyrrolidine (0.0495 g, 0.696 mmol), and acetic acid (0.0446 g, 0.743 mmol) were stirred in THF (5 mL) and heated to reflux overnight. When the reaction had cooled, water (5 mL) was added. The aqueous solution was extracted with 4 8-mL portions of ethyl acetate. The combined organic layers were dried with Na_2_SO_4_, and the solvent was removed with rotary evaporation.

5-hydroxypyridine-2-carboxaldehyde (0.0798 g, 0.648 mmol), methylsulfonylacetic acid (0.0875 g, 0.633 mmol), β-alanine (0.0569 g, 0.639 mmol) were stirred into THF (5 mL) and heated to reflux overnight. After the reaction had cooled water (5 mL) was added, and the aqueous layer was extracted with four 8-mL portions of ethyl acetate. The combined organic layers were rinsed with brine and dried with Na_2_SO_4_, and the solvent was removed by rotary evaporation. The two crude products were combined and eluted from silica using 80:20 ethyl acetate/hexanes. Rotary evaporation and high vacuum provided the product (0.086 g, 38% yield).

H-1 NMR (d_6_-acetone)

9.41 (s, 1H), 8.31 (d, J = 2.7, 1H), 7.61 (d, J = 8.4, 1H), 7.49, 7.38 (ABq, J = 15.2, 2H), 7.31 (dd, J = 8.4, 2.7, 1H), 3.07 (s, 3H).

C-13 NMR (d_6_-acetone)

154.98, 142.85, 140.94, 139.17, 128.45, 126.48, 122.14, 42.25.

HRMS calc. for C_8_H_9_NO_3_S (M-1) m/z 198.0225; found m/z 198.0239.

**Compound 5: (E)-2-(2-Methylsulfonyl)vinyl-5-nitrothiophene**

Lithium chloride (0.216 g, 3.84 mmol) was suspended in acetonitrile (20 mL) and stirred at room temperature. Sequentially were added DMeSUMP (0.587 g, 2.55 mmol) in acetonitrile (1 mL), DBU (0.324 g, 2.13 mmol) in a small portion acetonitrile, and 2-formyl-5-nitrothiophene (0.334 g, 2.13 mmol) in acetonitrile (1.4 ml); the last solution was added dropwise. After the reaction was stirred for 2 hr, it was quenched with a 6-mL portion of saturated ammonium chloride. After the volatiles were removed, a small portion of water was added, and the solution was extracted with 4 16-mL portions of DCM. After drying with MgSO_4_, the solvent was removed. The crude product was eluted from silica with 70:30 ethyl acetate/hexanes. Removal of the solvent by rotary evaporation and high vacuum provided a soft yellow solid (0.267 g, 53.8% yield).

H-1 NMR (CDCl_3_)

7.91 (d, J = 4.2, 1H), 7.68 (d, J = 15.3, 1H), 7.30 (d, J = 5.1, 1H), 6.95 (d, 15.3, 1H), 3.10 (s, 3H).

C-13 NMR (CDCl_3_)

163.51, 142.08, 134.69, 130.57, 129.443, 128.89, 43.17.

HRMS calc. for C_7_H_8_NO_4_S_2_ (M+1) m/z 233.9895; found m/z 233.9880.

**Compound 6: (E)-4-(2-Methylsulfonyl)vinylquinoline**

Lithium chloride (0.124g, 2.93 mmol) was suspended in acetonitrile (14 mL) at 25 °C. Successively were added DMeSUMP (0.368g, 1.50 mmol) in acetonitrile (1.2 mL), DBU (0.248 g, 1.63 mmol) in acetonitrile (1.0 mL), and 4-formylquinoline (0.252 g, 1.60 mmol) in acetonitrile (1.0 mL); the last solution was added dropwise. The reaction was followed to completion using TLC, then it was quenched with a 4-mL portion of saturated NH_4_Cl. After the volatiles were removed and a small portion of water added, the solution was extracted with four 12-mL portions of DCM. After the combined organic layers were dried with MgSO_4_, the solvent was removed by rotary evaporation. The product was eluted from silica with 75:25 ethyl acetate/methyl *tert*-butyl ether. Removal of the solvent by rotary evaporation and high vacuum provided the product (0.275 g, 74.8% yield).

H-1 NMR (CDCl_3_)

9.00 (d, J = 4.5, 1H), 8.42 (d, J = 15.3 Hz, 1H), 7.55 (d, J = 4.5, 1H), 7.19 (d, J = 15.3, 1H), 3.15 (s, 3H)

C-13 NMR (CDCl_3_)

149.87, 148.54, 138.77, 137.73, 132.70, 130.32, 127.98, 125.69, 123.00, 118.65, 43.02.

HRMS calc. for C_12_H_12_NO_2_S (M+1) m/z 234.0589; found m/z 234.0600.

**Compound 7: (E)-2-(2-Methylsulfonyl)vinylquinoline**

Lithium chloride (0.145 g, 3.42 mmol), was suspended in ACN and stirred at room temperature. Successively were added DMeSUMP (0.532 g, 2.30 mmol) in ACN (1.5 mL), DBU (0.305 g, 2.00 mmol) in ACN (1 mL), and 2-formylquinoline (0.301 g, 1.92 mmol) in ACN (2.4 mL); the last solution was added dropwise. The reaction was stirred for 1.5 hours and quenched with saturated NH_4_Cl (6 mL). After the volatiles were removed with rotary evaporation, and water (8 mL) was added. The aqueous layer was extracted with four 15-mL portions of DCM. The combined organic layers were dried with MgSO_4_, and the solvent was removed with rotary evaporation. The crude product was dissolved in a small portion of DCM and eluted from silica using 40:60 ethyl acetate/hexanes. Removal of the solvent by rotary evaporation and high vacuum provided a yellow solid (0.216 g, 48.5% yield).

H-1 NMR (CDCl_3_)

8.23 (d, J = 8.4, 1H), 8.10 (d, J = 8.7, 1H), 7.87-7.81 (m, 1H), 7.81, 7.65 (ABq, J_AB_ = 15.0, 2H) 7.80-7.73 (m, 1H), 7.63-7.56 (m, 1H), 7.54 (d, J = 8.4, 1H) 3.10 (s, 3H).

C-13 NMR (CDCl_3_)

150.72, 148.28, 142.75, 137.28, 131.76, 130.48, 129.94, 128.49, 127.92, 127.66, 121.69, 43.14.

HRMS calc. for C_12_H_12_NO_2_S (M+1) m/z 234.0589; found m/z 234.0600.

**Compound 8: (E)-5-(2-Methylsulfonyl)vinylisoquinoline**

Lithium chloride (0.153 g, 3.6 mmol), was suspended in ACN (16.5 mL) and stirred at room temperature. Successively were added DMeSUMP (0.530 g, 2.4 mmol) in ACN (1.2 mL), DBU (0.291 g, 2.2 mmol) in ACN (1 mL), and 5-formylisoquinoline (0.314 g, 2.00 mmol) in a small portion of ACN; the last solution was added dropwise. The reaction was stirred for 70 minutes and quenched with saturated NH_4_Cl (20 mL). After the volatiles were removed with rotary evaporation, and a small portion of water was added. The aqueous layer was extracted with four 15-mL portions of DCM. The combined organic layers were dried with MgSO_4_, and the solvent was removed with rotary evaporation. The crude product was eluted from silica using ethyl acetate. Removal of the solvent provided the product (0.383 g, 88.6% yield after the first column). The product was eluted from a fresh batch of silica with 50:50 2-propanol/hexanes. Rotary evaporation and high vacuum provided the product (0.116 g, 24.9% yield).

H-1 NMR (CDCl_3_)

9.35 (s, 1H), 8.67 (d, J = 6.0, 1H), 8.38 (d, J = 15.3, 1H), 8.13 (d, J = 8.4, 1H), 8.00 (d, J = 6.6, 1H), 7.96 (d, J = 6.3, 1H), 7.70 (m, 1H), 7.11 (d, J = 15.3, 1H), 3.14 (s, 3H).

C-13 NMR (CDCl_3_)

153.37, 144.39, 139.11, 134.03, 131.22, 129.68, 129.62, 128.76, 128.41, 126.93, 115.91, 43.23.

HRMS calc. for C_12_H_12_NO_2_S (M+1) m/z 234.0589; found m/z 234.0600.

**Compound 9: (E)-5-(2-Cyclopropylsulfonyl)vinylisoquinoline**

Lithium chloride (0.074 g, 1.7 mmol) was suspended in acetonitrile at 25 °C. Successively were added sulfonylphosphonate **18A** (0.271 g, 0.95 mmol) in acetonitrile (0.7 mL), DBU (0.145 g, 0.95 mmol) in acetonitrile (0.6 mL), and 5-formylisoquinoline (0.150 g, 0.95 mmol) in acetonitrile (4.0 mL); the last solution was added dropwise. The reaction was followed to completion using TLC then quenched with saturated NH_4_Cl. After the volatiles were removed and water (5 mL) was added, the solution was extracted with four 7-mL portions of DCM. After the combined organic layers were dried with MgSO_4_, the solvent was removed by rotary evaporation. The product was eluted from silica with 45:55 acetone:hexanes. Removal of the solvent by rotary evaporation and high vacuum provided the product (0.188 g, 76.3% yield).

H-1 NMR (CDCl_3_)

9.33 (s, 1H), 8.66 (d, J = 6.00, 1H), 8.29 (d, J = 15.3, 1H), 8.11 (d, J = 8.4, 1H), 8.00 (d, J = 7.2, 1H), 7.95 (d, J = 6.0, 1H), 7.69 (dd, J = 8.4, 7.2, 1H), 7.06 (d, J = 15.3, 1H), 2.56-2.46 (m, 1H), 1.42-1.33 (m, 2H), 1.18-1.08 (m, 2H).

C-13 NMR (CDCl_3_)

153.19, 144.04, 138.23, 134.08, 130.98, 129.69, 129.24, 128.86, 128.74, 31.26, 5.53.

HRMS calc. for C_14_H_14_NO_2_S (M+1) m/z 260.074526; found m/z 260.0803.

**Compound 10: 4-(2-pyridylsulfonyl)vinylpyridine**

**10A** Diethyl 2-pyridylthiomethylphosphonate

2-Mercaptopyridine (1.03 g, 9.27 mmol), Cs_2_CO_3_ (3.072 g, 9.43 mmol), and Diethyl (*p*-toluenesulfonyloxymethyl)phosphonate (2.51 g, 7.79 mmol) were stirred with DMF (1 mL) and heated to 80 °C for 4.5 hours. The mixture was dissolved in ethyl acetate (60 mL), extracted twice with 5% LiCl (60 mL) and extracted twice with brine. The organic layer was dried with Na_2_SO_4_, and the solvent was removed by rotary evaporation. The crude product was eluted from silica using 90:10 ethyl acetate/hexanes then ethyl acetate. Removal of the solvent with rotary evaporation and high vacuum gave the product (1.12 g, 57.7%).

H-1 NMR (CDCl_3_)

8.49-8.42 (m, 1H), 7.58-7.47 (m, 1H), 7.32-7.21 (m, 1H), 7.08-6.99 (m, 1H), 4.23-4.07 (m, 4H), 3.69 (d, J = 13.5, 2H), 1.30 (t, J = 6.9, 6H).

C-13 NMR (CDCl_3_)

149.25, 136.15, 129.48, 122.25, 119.97, 62.66 (d, J = 6.3), 29.78 (d, J = 120.2), 16.33 (d, J = 6.1).

P-31 NMR (CDCl_3_)

24.00

**10B** Diethyl 2-pyridylsulfonylmethylphosphonate

Thioether 10A (1.10 g, 4.21 mmol) was dissolved in 30 mL methanol and 30 mL of water was added. Oxone (26.012 g, 42.2 mmol) was added, and the reaction was stirred for two days. The solvent was removed by rotary evaporation. After DCM (100 mL) was added, the mixture was filtered through celite, and water was added (20 mL). The water layer was extracted using 2 50-mL portions of DCM, and the combined DCM layers were washed with 3 100-mL portions of brine. The organic phase was dried with Na_2_SO_4_, and the solvent was removed with rotary evaporation. The crude product was eluted from silica with 85:15 ethanol/ethyl acetate. Removal of the solvent provided the product (0.69 g, 56% yield).

H-1 NMR (CDCl_3_)

8.28 (d, J = 5.4, 1H), 8.12 (dd, J = 7.8,2.4, 1H), 7.58-7.41 (m, 2H), 4.51 (d, J = 16.2, 2H), 4.24-4.07 (m, 4H), 1.32 (t, J = 7.2, 6H).

C-13 NMR (CDCl_3_)

147.00, 140.89, 129.82, 127.90, 125.56, 63.54 (d, J = 6.1), 49.71 (d, J = 136.7), 16.23 (d, J = 6.2 Hz).

P-31 NMR (CDCl_3_)

10.82.

**10** 4-(2-(2-Pyridyl)sulfonyl)vinylpyridine

Lithium chloride (0.0661 g, mmol) was suspended in acetonitrile (8.5 mL) and stirred at room temperature. Successively were added diethyl 2-pyridylsulfonylmethylphosphonate 10B (0.300 g, mmol) in acetonitrile (0.70 mL), DBU (0.1297g, mmol) in acetonitrile (1 mL), and 4-formylpyridine (0.0912 g, mmol) were added; the last solution was added dropwise. The reaction was stirred for two hours then quenched with saturated NH_4_Cl (2.3 mL). After the volatiles were removed with rotary evaporation, a small portion of water was added, and this solution was extracted with four 7-mL portions of DCM. After the combined organic layers were dried with MgSO_4_, the solvent was removed with rotary evaporation. The crude product was eluted from silica with 85:15 ethyl acetate/ethanol. Removal of the solvent provided the product (0.18 g, 86% yield).

H-1 NMR (CDCl_3_)

8.78-8.64 (m, 2H), 8.27-8.22 (m, 1H), 8.16-8.08 (m, 1H), 7.80-7.68 (m, 2H), 7.55-7.36 (m, 4H).

C-13 NMR (CDCl_3_)

150.89, 148.10, 144.69, 140.97, 139.40, 129.79, 129.33, 127.04, 125.42, 122.34.

Compound 10 did not ionize during attempted MS analysis.

**Compound 11: (E)-4-(2-Morpholinosulfonyl)vinylpyridine**

**11A** Methylsulfonylmorpholine

To a solution of morpholine (1.000 g, 11.5 mmol) in DCM (20 mL) was added TEA (1.161 g, 11.5 mmol), and the mixture was stirred at -10 °C. Methanesulfonyl chloride (1.972, 17.2 mmol) was added dropwise. The reaction mixture was stirred for 6 hr at 0° C and checked for completion by TLC. The reaction was quenched with saturated NH_4_Cl (3 mL), and the aqueous solution was extracted with 3 10-mL portions of DCM. The combined organic layers were successively washed with water and brine. The organic layers were dried with Na_2_SO_4_, and the solvent was removed with rotary evaporation. The product was eluted from silica with 45:55 to 50:50 ethyl acetate/hexanes. Removal of the solvent with rotary evaporation and high vacuum provided the product (1.605 g, 84.5% yield).

H-1 NMR (CDCl_3_)

3.82-3.79 (m, 4H), 3.25-3.22 (m, 4H), 2.82 (s, 3H).

C-13 NMR (CDCl_3_)

66.33, 45.88, 33.96.

**11B** Diethyl morpholinosulfonylmethylphosphonate

Methylsulfonylmorpholine **11A** (0.901 g, 5.45 mmol) was dissolved in THF (30 mL) at -70 °C with stirring. A solution of Lithium hexamethyldisilazane (10.9 mL, 10.9 mmol) in THF was added dropwise and the reaction stirred for an hour. Diethyl chlorophosphate (0.940 g, 5.44 mmol) in THF (13.8 mL) was added dropwise and the reaction stirred for a few minutes. The cooling bath was removed, and the reaction was stirred overnight at room temperature. Water (2.5 mL) and ethyl acetate (25 mL) were added. This mixture was extracted with saturated Na_2_CO_3_ (25 mL), saturated NH_4_Cl (25 mL), and brine (25 mL). After the organic layer was dried with Na_2_SO_4_, the volatiles were removed with rotary evaporation. The product was eluted from silica using 90:10 ethyl acetate/ethanol. Removal of the solvent by rotary evaporation and high vacuum provided the solid (0.775 g, 47.3% yield).

H-1 NMR (CDCl_3_)

4.29-4.19 (m, 4H), 3.78-3.75 (m, 4H), 3.53 (d, J = 17.4, 2H), 3.34 (m, 4H), 1.38 (t, J = 7.1, 6H)

C-13 NMR (CDCl_3_)

66.49, 63.56 (d, J = 6.5), 46.30 (d, J = 139.4), 45.96, 16.36 (d, J = 6.5).

P-31 NMR (CDCl_3_)

12.89.

**11** (E)-4-(2-Morpholinosulfonyl)vinylpyridine

Lithium chloride (0.090 g, 2.12 mmol) was suspended in acetonitrile (9 mL) at 25 °C. Successively were added Diethyl morpholinosulfonylmethylphosphonate **11B** (0.352 g, 1.17 mmol) in acetonitrile (0.9 mL), DBU (0.178 g, 1,17 mmol) in acetonitrile (0.6 mL), and 4-formylpyridine (0.125 g, 1.17 mmol) in acetonitrile (2 mL); the last solution was added dropwise. The reaction was stirred until judged complete by TLC, then quenched with a 3-mL portion of saturated NH_4_Cl. After the volatiles were removed and a small portion of water added, the solution was extracted with four 9-mL portions of DCM. After the combined organic layers were dried with Na_2_SO_4_, the solvent was removed by rotary evaporation. The product was eluted from silica with 98:2 ethyl acetate/ethanol. Removal of the solvent by rotary evaporation and high vacuum provided the product (0.213 g, 89.9%).

H-1 NMR (CDCl_3_)

8.78-8.67 (m, 2H), 7.44 (d, J = 15.6, 1H), 7.41-7.36 (m, 2H), 6.90 (d J = 15.6, 1H), 3.82-3.79 (m, 4H), 3.26-3.23 (m, 4H).

C-13 NMR (CDCl_3_)

150.66, 140.99, 139.87, 126.03, 121.99, 66.29, 45.67.

HRMS calc. for C_11_H_15_N_2_O_3_S (M+1) m/z 255.08034; found m/z 255.0786.

**Compounds 12: 3-Methylsulfonylacrylamide and 13: 3-Phenylsulfonylacrylamide**

**12A** 2,3-Dibromopropanamide

Acrylamide (1.020 g, 14.35 mmol) was dissolved in methanol (6 mL) and stirred. Bromine (2.25 g, 14.08 mmol) was added dropwise. The reaction was refluxed for 2.5 hours. The solvent was removed with rotary evaporation. The product was recrystallized from ethanol and dried under a gentle stream of N_2_ to provide the solid (0.620 g, 19%).

H-1 NMR (d_6_-DMSO)

7.88 (s, 1H), 7.46 (s, 1H), 4.61-4.50 (m, 1H), 3.97-3.79 (m, 2H).

C-13 NMR (d_6_-DMSO)

168.36, 44.58, 31.86.

**12** 3-Methylsulfonylacrylamide

2,3-Dibromopropanamide **12A** (0.403 g, 1.74 mmol) was dissolved in DMF (5.4 mL), and sodium methylsulfinate (0.200 g, 1.96 mmol) in water (1.3 mL) was stirred in. The mixture was heated to 80 °C for about 22 hours. After addition of toluene, the volatiles were removed with rotary evaporation. The product was eluted from silica using 90:10 ethyl acetate/ethanol, and the solvent removed by rotary evaporation and high vacuum. The partially purified product was eluted from fresh silica using 98:2 ethyl acetate/ethanol. Removal of the solvent by rotary evaporation provided the product (0.059 g, 11% yield).

H-1 NMR (d_6_-acetone)

7.42 (d, J = 15.0, 1H), 7.00 (d, J = 15.0, 1H), 3.10 (s, 3H).

C-13 NMR (d_6_-acetone)

163.15, 139.65, 134.80, 41.50.

HRMS calc. for C_4_H_6_NO_3_S (M-1) m/z 148.006841; found m/z 148.0048.

**13** 3-Phenylsulfonylacrylamide

2,3-Dibromopropanamide **12A** (0.403 g, 1.74 mmol) and sodium phenylsulfinate (0.430 g, 2.62 mmol) were dissolved in DMF (3.5 mL) and heated to 80 °C for 12 hours. Heptane was added, and the volatiles were removed by rotary evaporation and high vacuum. The product was dissolved in water (9 mL), and it was extracted three times with 6-mL portions of ethyl acetate. The combined organic layers were dried with Na_2_SO_4_, and the solvent was removed with rotary evaporation. The product was eluted from silica using ethyl acetate. Removal of the solvent by rotary evaporation and high vacuum provided the product (0.113 g, 30.8% yield).

H-1 NMR (d_6_-DMSO)

8.04 (broad s, 1H), 7.95-7.56 (m, 6H), 7.46 (d, J = 15.0, 1H), 6.99 (d, J = 15.0, 1H).

C-13 NMR (d_6_-DMSO)

163.53, 139.49, 139.35, 135.83, 134.83, 130.30 128.17.

HRMS calc. for C_9_H_10_NO_3_S (M+1) m/z 212.038141; found m/z 212.0379.

**Compound 14: (E)-2-(Methylsulfonyl)vinylalanine hydrochloride**

**14A** Boc-Asp(NCH_3_(OCH_3_))-OtBu.

To a solution of Boc-Asp(OH)-OtBu (2.52 g, 8.710 mmol), HBTU (3.34 g, 8.807 mmol), and N,O-dimethylhydroxylamine hydrochloride (0.84 g, 8.612 mmol) in DMF (85 mL) was added DIPEA (3.4 g, 4.5ml) over a period of 15 minutes. The resulting mixture was stirred at room temperature for 6 hours. The result was extracted into a 170-mL portion of ethyl acetate, rinsed 2x with 170-mL aliquots of 5% LiCl solution, and 1x with brine (85 mL). The organic layer was dried over Na_2_SO_4_ and the volatiles were removed with rotary evaporation and high vacuum. The crude product was purified eluted from silica with 50:50 ethyl acetate/hexanes, and the solvent was removed by rotary evaporation and high vacuum. The yield was 1.781 g (62% yield).

H-1 NMR (CDCl_3_)

5.86 (d, J = 2.3, 2H), 4.48 (m, 1H), 3.71 (s, 3H), 3.19 (s, 3H), 3.1-2.7 (m, 2H), 1.48 (s, 9H), 1.46 (s, 9H).

C-13 NMR (CDCl_3_)

171.81, 170.58, 155.78, 81.77, 79.52, 61.24, 50.39, 34.72, 32.01, 28.36, 27.92.

**14B** Boc-Asp(H)-OtBu

Boc-Asp(NCH_3_(OCH_3_))-OtBu, (2.38 g, 7.17 mmol) was dissolved in a 31-mL portion of dry THF and cooled to -75 °C. A solution of Dibal-H (11 mL, 11 mmol) in hexanes was added dropwise over 45 min and the reaction stirred for 3.5 hours. The reaction was monitored by microextraction and TLC visualized with DNP. The reaction mixture was poured into a separatory funnel containing a 5% solution of KHSO_4_ (137 mL) and a 110-mL portion of diethyl ether. The aqueous layer was extracted three 65-mL portions of ether, and the combined ether layers were washed with three portions each of 1 M HCl, 1 M NaHCO_3_, and brine. The organic layer was dried with Na_2_SO_4_, and the solvent was removed with rotary evaporation and high vacuum. The crude product was eluted from silica with 30:70 ethyl acetate/hexanes. The yield was 1.29 grams (66%).

H-1 NMR (CDCl_3_)

9.75 (s, 1H), 5.38 (d, J = 6.6, 1H), 4.50 (m, 1H), 3.07-2.91 (m, 2H), 1.47 (s, 9H), 1.46 (s, 9H).

C-13 NMR (CDCl_3_)

199.41, 169.94, 155.37, 82.72, 49.32, 46.39, 28.30, 27.87.

**14C** Boc-Ala(CH_2_CH=CHSO_2_CH_3_)-OtBu

LiCl (0.119 g, 2.81 mmol) was suspended in a 15-mL portion of ACN, and a solution of DMeSUMP (0.408 g, 1.772 mmol) in a 0.60-mL portion of ACN was added at room temperature. A solution of DBU (0.239 g, 1.570 mmol) in a small portion of ACN was added. A solution of Boc-Asp(H)-OtBu **14B** 0.392 g, 1.434 mmol) in a 1-mL portion of ACN was added dropwise over eight minutes. The reaction was monitored to completion by TLC using PMA and KMnO_4_ stains. The reaction was quenched with saturated NH_4_Cl; the solvent was removed by rotary evaporation; and a small portion of water added. The water layer was extracted four times with 12-mL portions of DCM. The combined DCM layers were dried with MgSO_4_, and the solvent was removed with rotary evaporation and high vacuum. The crude product was eluted from silica using 40:60 ethyl acetate/hexanes. The yield was 0.363 g (73%).

H-1 NMR (CDCl_3_)

6.95 (m, 1H), 6.49 (d, J = 15.3, 1H), 5.21 (d, J = 6.6, 1H), 4.37 (m, 1H), 2.94 (s, 3H), 2.85-2.61 (m, 2H), 1.49 (s, 9H), 1.46 (s, 9H).

C-13 NMR (CDCl_3_)

169.90, 155.03, 142.81, 132.38, 83.21, 52.70, 42.80, 34.84, 28.30.

**14** (E)-2-(Methylsulfonyl)vinylalanine hydrochloride.

The protected amino acid **14C** (0.363 g, 1.04 mmol) was dissolved in a 1.2 mL portion of dry DCM at 0 °C, and a 6-mL portion of trifluoroacetic acid was added over 15 minutes. The reaction was stirred overnight at room temperature, and the volatiles were removed by rotary evaporation and high vacuum. In some syntheses the crude product was suspended in toluene, which was then removed by rotary evaporation. A column having 20 equivalents of Dowex 50 in the H^+^ form was prepared. The crude product was dissolved in 0.5 column volumes (CV) of H_2_O, and the pH was checked. The solution was applied to the column, followed by two CVs of water. The product was eluted with 2 CV of 4 M HCl. The HCl solution was removed by rotary evaporation to yield a 0.115-gram portion of the hydrochloride salt 0.115 g (48 %).

H-1 NMR (D_2_O)

6.91-6.77 (m, 2H), 4.22 (m, 1H), 3.06 (s, 3H), 3.04-2.84 (m, 2H)

C-13 NMR (D_2_O)

170.77, 141.26, 132.67, 51.53, 41.56, 31.59.

HRMS calc. for C_6_H_12_NO_4_S (M+1) m/z 194.0487; found m/z 194.0492.

**Compound 15: (E)-2-(N,N-dimethylsulfonamido)vinylalanine hydrochloride**

**15A** Diethyl N,N-dimethylsulfamidomethylphosphonate

A solution of N,N-dimethyl methanesulfonamide (0.503 g, 4.1 mmol) was prepared in dry THF and cooled to -70 °C. A solution of *n*-BuLi (2.8 mL, 4.5 mmol) 1.6 M in hexanes was added dropwise with stirring over 15 minutes. The reaction was brought to -40 °C for two hours then returned to -70 °C. A solution of diethyl chlorophosphate (0.700 g. 4.1 mmol) in THF (4.1 mL) was added dropwise. The reaction was allowed to come to room temperature and stirred for several hours before being stored at 5 °C overnight. To the reaction mixture was added a 15-mL aliquot of water, and the product was extracted with four 15-mL portions of DCM, drying the organic layer with sodium sulfate. The volatiles were removed, and the crude product was purified over silica using 70:30 to 100:0 ethyl acetate/hexanes to provide 15A (0.319 g, 30%)

H-1 NMR

4.26 (4H, m), 3.56 (2H, d, J = 17.1), 2.96 (6H, s), 1.40 (t, J = 7.2).

C-13 NMR

63.44 (d, J = 6.6) 45.54 (d, J = 139.8), 37.65 (s), 16.34 (d, J = 6.3).

P-31 NMR

13.3 ppm (s).

**15B** Boc-Ala(CH_2_CH=CHSO_2_N(CH_3_)_2_)-OtBu

Lithium chloride (0.0794 g, 1.87 mmol) was suspended in acetonitrile (8 mL) at room temperature. Successively were added diethyl N,N-dimethylsulfonamidomethylphosphonate **15A** (0.259 g, 0.998 mmol) in ACN (1 mL), DBU (0.138 g, 0.907 mmol) in acetonitrile (1 mL), and Boc-Asp(H)-OtBu **14B** (0.343 g, 0.907 mmol) in acetonitrile (1.7 mL). The reaction was stirred until judged complete by TLC. After the reaction was quenched with saturated NH_4_Cl, the volatiles were removed, and a small portion of water was added. After this solution was extracted with four 10-mL portions of DCM. The combined organic layers were dried with MgSO_4_, and the solvent was removed with rotary evaporation. The product was eluted from silica with 35:65 ethyl acetate/hexanes. The solvent was removed with rotary evaporation and high vacuum to provide the product (0.186 g, 54.2% yield).

H-1 NMR (CDCl_3_)

6.67 (m, 1H), 6.21 (d, J = 15.3, 1H), 5.25-5.15 (m, 1H), 4.42-4.31 (m, 1H), 3.15-2.98 (m, 1H), 2.78 (s, 6H), 2.73-2.58 (m, 1H), 1.50 (s, 9H), 1.47 (s, 9H).

C-13 NMR (CDCl_3_)

170.05, 155.02, 141.52, 126.40, 82.78, 80.23, 52.86, 37.52, 34.84, 28.32, 28.04.

**15** (E)-2-(N,N-dimethylsulfonamido)vinylalanine hydrochloride

The protected amino acid **15B** was stirred in DCM (0.5 mL) at 0 °C, and TFA was added dropwise. The reaction was stirred for several hours at room temperature. Removal of the volatiles with rotary evaporation, two additions of toluene, each followed by rotary evaporation, and finally high vacuum provided the crude product. The crude product was dissolved in H_2_O (6 mL) and applied to a column of Dowex50-H^+^ (5.5 mL). The column was rinsed with H_2_O (8 mL); the load and wash were collected separately. The product was eluted with 2 M HCl (22 mL). The solvent was removed with rotary evaporation and high vacuum to provide the product (0.109 g, 85.6% yield).

H-1 NMR (d_6_-DMSO)

13.9 (broad s, 1H), 8.63 (s, 3H), 6.69-6.59 (m, 2H), 4.19 (broad s, 1H), 2.91-2.81 (m, 2H), 2.73-2.61 (m, 6H).

C-13 NMR (d_6_-DMSO)

170.43, 140.76, 127.20, 51.23, 37.69, 32.19.

HRMS calculated for C_7_H_15_N_2_O_4_S (M+1) m/z 223.0753; found m/z 223.0733.

**Compound 16: (E)-2-(Benzylsulfonyl)vinylalanine hydrochloride**

**16A** Diethyl benzylthiomethylphosphonate

Diethyl iodomethylphosphonate (1.017 g, 3.66 mmol) was dissolved in a 2-mL portion of acetone and stirred at room temperature under reduced light. Benzylthiol (0.853 g, 10.6 mmol) was dissolved in a 2.5-mL portion of acetone and added dropwise. K_2_CO_3_ was added, and the reaction refluxed for several days with monitoring by TLC and P-31 NMR. The volatiles were removed with rotary evaporation, and the product dissolved in water (36 mL). The aqueous layer was extracted with three 36-mL portions of ethyl acetate. The combined organic layers were extracted with brine (18 mL), saturated NaHCO_3_ (18 mL), 1 M HCl (18 mL), and brine (18 mL). The organic layer was dried with Na_2_SO_4_, and the solvent was removed with rotary evaporation. The crude product was eluted with ethyl acetate/hexane, 45:55 to 60:40, and the solvent was removed by rotary evaporation and high vacuum. The product mass was 0.610 grams (60.8% yield).

H-1 NMR (d_6_-acetone)

7.43-7.19 (m, 5H), 4.18-4.03 (m, 4H), 3.96 (s, 2H), 2.61 (d, J = 12.9, 2H), 1.28 (t, J = 6.9, 6H).

C-13 NMR (d_6_-acetone)

137.91, 129.25, 128.44, 127.11, 62.05 (d, J = 6.8), 36.35 (d, J = 5.3) 23.43 (d, J = 149.4), 16.00 (d, J = 5.6).

P-31 NMR (d_6_-acetone)

23.9.

**16B** Diethyl benzylsulfonylmethylphosphonate

Diethyl benzylthiomethylphosphonate 16A (0.610 g, 2.22 mmol) was dissolved in an 8.8-mL portion of methanol and stirred. Oxone (2.053 g, 3.34 mmol) was dissolved in an 8.8-mL portion of water and added dropwise. The reaction was stirred for two days and monitored to completion by TLC and P-31 NMR. Methanol was removed by rotary evaporation and a small portion of water was added to dissolve the salts. The aqueous layer was extracted with three 20-mL portions of DCM, and the combined organic layers were dried with Na_2_SO_4_. DCM was removed by rotary evaporation and high vacuum to yield 0.607 g of product (89.1% yield.

H-1 NMR in CDCl_3_

7.58-7.40 (m, 5H), 4.63 (s, 2H), 4.33-4.22 (m, 4H), 3.39 (d, J = 16.2, 2H), 1.41 (t, J = 7.2, 6H).

C-13 NMR in CDCl_3_

131.04, 129.20, 129.07, 128.12, 63.74 (d, J = 6.8), 60.28, 47.92 (d, J = 140), 16.34 (d, J = 6.0).

P-31 NMR in CDCl_3_

11.85.

**16C** Boc-Ala(CH_2_CH=CHSO_2_CH_2_Ph)-OtBu

LiCl (0.0566g, 1.335 mmol) was suspended in a 7.1-mL portion of acetonitrile. Diethyl benzylsulfonylmethylphosphonate 16B (0.259 g, 0.846 mmol) was dissolved in a 0.27-mL portion of acetonitrile and added. DBU (0.111 g, 0.732 mmol) was dissolved in a 0.45-mL portion of acetonitrile and added. Boc-Asp(H)-OtBu **14B** (0.200 g, 0.846 mmol) was dissolved in a 0.47-mL portion of acetonitrile and added dropwise. The reaction was monitored by P-31 NMR and by TLC using PMA and KMnO_4_ stains, and it was quenched by addition of a 3.8-mL portion of saturated ammonium chloride solution. Volatiles were removed by rotary evaporation, and a small portion of water was added. The aqueous layer was extracted with four 6-mL portions of DCM. The DCM layer was dried with MgSO_4_, and the DCM was removed by rotary evaporation. The crude product was eluted with 4-5% EtOAc in DCM. After removal of the solvent by rotary evaporation and high vacuum, the mass was 0.150 g (48%).

H-1 NMR (CDCl_3_)

7.39-7.34 (m, 5H), 6.69-6.62 (m, 1H), 6.26 (d, J = 15.3, 1H), 5.08 (d, J = 6.9, 1H), 4.36-4.27 (m, 1H), 4.23 (s, 2H), 2.72-2.62 (m, 2H), 1.48 (s, 9H), 1.47 (s, 9H).

C-13 NMR (CDCl_3_)

169.79, 144.47, 130.89, 130.14, 128.99, 128.90, 127.90, 83.18, 80.31, 61.37, 52.67 (broad), 34.76, 28.32, 27.96.

**16** (E)-2-(Benzylsulfonyl)vinylalanine hydrochloride.

Boc-Ala(CH_2_CH=CHSO_2_CH_2_Ph)-OtBu **16C** was dissolved in DCM (0.25 mL) at 0 °C, and TFA (1.0 mL) was added dropwise. The reaction was stirred for about twelve hours, and the volatiles were removed with rotary evaporation and high vacuum. A column of Dowex 50 in twenty-fold excess was prepared. The crude product was dissolved in 0.5 CV of water and applied to the column. The column was eluted with 1.5 CV of water and the first pool was collected as the load and wash. The product was eluted with 6 CV of 2 M HCl and 6 CV of 4 M HCl. The aqueous volatiles were removed with rotary evaporation and high vacuum. A waxy solid was obtained 0.068 g (44% yield).

H-1 NMR (d_6_-DMSO)

7.43-7.33 (m, 5H), 6.79-6.52 (m, 2H), 4.48 (s, 1H), 4.15 (t, J = 6.2, 1H), 2.89-2.74 (m, 2H).

C-13 NMR (d_6_-DMSO)

170.32, 142.03, 132.34, 131.64, 128.95, 128.89, 128.81, 59.97, 51.00, 32.07.

HRMS calc. for C_12_H_14_NO_4_S (M-1) m/z 268.0643; found m/z 268.0655.

**Compound 17: (E)-2-(Isopropylsulfonyl)vinylalanine hydrochloride**

**17A** Diethyl acetylthiomethylphosphonate

Diethyl iodomethylphosphonate (1.50 g, 5.40 mmol), Tetrabutylammonium iodide (0.199 g, 0.540 mmol), and Potassium thioacetate (0.677 g, 5.93 mmol) were stirred with THF (9 mL) and heated to a bath temperature of 70 °C for several hours with monitoring by P-31 NMR. The THF was removed by rotary evaporation, and the mixture was dissolved in ethyl acetate. The mixture was eluted from silica (3 g), and the ethyl acetate was removed by rotary evaporation and high vacuum to provide the product (1.128 g, 92.5% yield).

H-1 (CDCl_3_)

4.20-4.08 (m, 2H), 3.23 (d, J= 13.8, 2H), 2.40 (s, 3H), 1.33 (t, J = 7.2, 3H).

C-13 NMR (CDCl_3_)

193.08, 62.80 (d, J = 6.5), 30.07, 22.26 (d, J = 151.4), 16.38 (d, J = 6 Hz).

P-31 NMR (CDCl_3_)

22.78.

**17B** Diethyl thiomethylphosphonate

Diethyl acetylthiomethylphosphonate **17A** (1.128 g, 4.99 mmol) was stirred with methanol (0.9 mL). Potassium hydroxide (10 M, 0.75 mL) was added, and the reaction stirred at room temperature. The reaction was monitored using P-31 NMR. Volatiles were removed with rotary evaporation, and the mixture was brought to pH ~1 using HCl (1 M, 18 mL). The aqueous layer was extracted with four 20-mL portions of DCM, and the organic layer was dried with Na_2_SO_4_. The DCM was removed with rotary evaporation to provide the product (0.676 g, 73.7% yield).

H-1 NMR (CDCl_3_)

4.25-4.11 (m, 4H), 2.69 (dd, J = 13.2, 8.1, 2H), 1.86 (q, J = 8.4, 1H) 1.36 (t, J = 7.1, 6H).

C-13 NMR (CDCl_3_)

63.08 (d, J = 6.7), 17.66 (d, J = 125.7), 16.46 (d, J = 6.0).

P-31 NMR (CDCl_3_)

24.70.

**17C** Diethyl isopropylthiomethylphosphonate

A solution of diethyl thiomethylphosphonate **17B** (2.2 mmol), 2-iodopropane (2.4 mmol), and potassium carbonate (2.6 mmol) in a 0.62 mL-aliquot of DMF were placed in a vial under N_2_ and stirred at 60 °C for 15 hours with monitoring by P-31 NMR. The crude product was dissolved in a 6-mL portion of water and extracted with 10-mL portions of DCM three times. The combined organic layers were extracted against a 10-mL portion of 3 M NaOH, a 10-mL portion of brine, dried with Na_2_SO_4_, and the volatiles removed with rotary evaporation. The crude product was eluted from silica using 80:20 EtOAc/hexanes (side products eluted with 100% EtOAc), and the volatiles were removed with rotary evaporation and high vacuum. The yield of diethyl isopropylthiomethylphosphonate was 0.370 g (74%).

H-1

4.19 (m, 4H), 3.19 (septet, J = 6.6, 1H), 2.79 (d, J = 14.4, 2H), 1.37 (t, J = 6.9, 6H), 1.30 (d, J = 6.6, 6H)

C-13

62.63 (d, J = 6.7), 36.20 (d, J = 4.4), 24.19 (d, J = 149.4), 22.81 (s), 16.48 (d, J = 6.0).

P-31

24.64 (s)

**17D** Diethyl isopropylsulfonylmethylphosphonate

To a solution of diethyl isopropylthiomethylphosphonate **17C** (0.36 g) in methanol was added a solution of Oxone (1.47 g) in water dropwise with stirring at 20 °C. The reaction was stirred overnight, and the volatiles were removed. The product was dissolved in 5 mL of water, extracted with four 10-mL portions of ethyl acetate, dried with sodium sulfate, and the volatiles were removed, leaving a white solid. The product (0.303 g, 73%) was used without further purification

H-1

4.26 (m, 4H), 3.72 (septet, J = 6.9, 1H), 3.58 (d, J = 16.8, 2H), 1.43 (d, J = 6.9, 6H) 1.39 (t, J = 7.2, 6H).

C-13

63.66 (d, J = 6.6), 54.19 (s), 47.25 (d, J = 139.2), 16.33 (d, J = 6.5), 15.18 (s).

P-31

12.07 (s).

**17E** Boc-Ala(CH_2_CH=CHSO_2_iC_3_H_7_)-OtBu

Lithium (0.064 g, 1.50 mmol) was suspended in acetonitrile (6 mL) with stirring at room temperature. Successively were added diethyl isopropylsulfonylmethylphosphonate **17D** (0.232 g, 0.906 mmol) in acetonitrile (0.7 mL), DBU (0.132 g, 0.867 mmol) in acetonitrile (0.7 mL), and Boc-Asp(H)-OtBu **14B** (0.225 g, 0.823 mmol) in acetonitrile (1.2 mL); the last solution was added dropwise. The reaction was stirred for 3 hours and quenched with saturated ammonium chloride (3 mL). After the volatiles were removed, a small portion of water was added. The solution was extracted with four 5-mL portions of DCM, and the combined organic layers were dried with MgSO_4_. The solvent was removed by rotary evaporation. The compound was eluted from silica with 35:65 ethyl acetate/hexanes; removal of solvent by rotary evaporation provided a white solid (0.105 g, 34%).

**17** (E)-2-(Isopropylsulfonyl)vinylalanine hydrochloride

The protected amino acid **17E** (0.105 g, 0.278 mmol) was dissolved in a 0.33-mL portion of DCM. TFA was added dropwise. The reaction was stirred overnight at room temperature. The volatiles were removed with rotary evaporation followed by stripping with toluene (3X) and high vacuum. The sample was dissolved in DI water (pH 1.8) and applied to a 6-mL column Dowex-50. After 2 column volumes of water were applied, the sample was eluted with 4 column volumes of 2 M HCl. The volatiles were removed by rotary evaporation, followed by high vacuum to leave 0.097 g product. The yield was quantitative.

H-1 (DMSO)

13.9 (s, 1H), 8.67 (s, 3H), 6.78 (m, 2H), 4.19 (m, 1H), 3.17 (septet, J = 6.9, 1H) 2.88 (m, 2H), 1.20 (d, J= 6.9, 6H).

HRMS calc. for C_8_H_16_NO_4_S (M+1) m/z 222.0800; found m/z 222.0787.

**Compound 18: (E)-2-(Cyclopropylsulfonyl)vinylalanine hydrochloride**

**18A** Diisopropyl cyclopropylsulfonylmethylphosphonate

Diisopropyl bromomethylphosphonate (0.615 g, 2.37 mmol), sodium S-cyclopropylsulfinate (0.452 g, 3.53 mmol), and tetrabutylammonium iodide (0.0910 g, 0.246 mmol) were dissolved in a 3.0 mL-aliquot of DMF. The mixture was stirred at 92 °C for 13 hours and monitored via P-31 NMR. The mixture was dissolved in a 14-mL aliquot of ethyl acetate and was extracted once against a 7-mL aliquot of 5% aqueous LiCl and once against brine. The residual DMF was removed by rotary evaporation with heptane. The organic layer was dried with sodium sulfate. The crude product was eluted from 10 grams of silica using 70:30 ethyl acetate/hexanes. Removal of the solvent by rotary evaporation and high vacuum gave the product (0.329 g, 49%).

H-1 NMR

4.87 (m, 2H), 3.59 (d, J = 16.8, 2H), 3.04 (m, 1H), 1.41 (d, J = 6.3, 6H), 1.40 (d, J = 6.0, 6H) 1.33 (m, 2H), 1.11 (m, 2H).

C-13 NMR

72.78 (d, J = 6.6), 52.37 (d, J = 140.4), 31.44 (s), 24.17 (d, J = 3.5), 23.83 (d, J = 5.5), 5.58 (s).

P-31 NMR

10.04 (s).

**18B** Boc-Ala(CH_2_CH=CHSO_2_C_3_H_5_)-OtBu

To a stirred solution of LiCl (0.90 g, 2.12 mmol) in ACN (9 mL) was added diisopropyl S-cyclopropylsulfonylmethylphosphonate **18A** (0.319 g, 1.12 mmol) in ACN (1 mL), and DBU (0.178 g, 1.12 mmol) in ACN (1 mL) at room temperature under N_2_. To this solution was added Boc-Asp(H)-OtBu **14B** (0.299 g, 1.09 mmol) in ACN (2 mL) dropwise. All three addition flasks were rinsed with small portions of ACN. The reaction was monitored by TLC, staining with PMA and KMnO_4_. The reaction was quenched with saturated NH_4_Cl (3 mL) after 3.5 hours, and the volatiles were removed with rotary evaporation. The product was dissolved in a small portion of water and extract with four 5-mL portions of DCM. The combined organic layers were dried with Na_2_SO_4_, and the volatiles were removed. The dry-loaded product was eluted from silica using 30:70 EtOAc/hexanes and fractions were monitored with KMnO_4_ and PMA staining. The solvent was removed from pooled fractions using rotary evaporation and high vacuum to provide the product (0.161 g, 39% yield).

H-1 NMR

6.81 (m, 1H), 6.45 (m, 1H), 5.22 (m, 1H), 4.38 (M, 1H), 2.87-2.63 (m, 2H), 2.35 (m, 1H), 1.50 (s, 9H), 1.470 (s, 9H), 1.27 (m, 4H),

C-13 NMR

169.98 (s), 155.02 (s), 141.73 (s), 131.70, 83.13, 80.23, 52.74, 34.18, 30.90, 28.31, 28.03, 5.25

**18** (E)-2-(Cyclopropylsulfonyl)vinylalanine hydrochloride

The protected amino acid **18B** (0.147 g, 0.392 mmol) was dissolved in a 0.5 mL portion of DCM under nitrogen. TFA (2.0 mL) was added dropwise at 0 °C. The reaction came to room temperature and was stirred overnight. The solvent was removed with rotary evaporation, and two small portions of toluene were added and removed under reduced pressure. The crude product was put under high vacuum. The sample was dissolved in a 3-mL portion of deionized water and loaded onto a 6-mL column of rinsed Dowex-50H^+^. The column was rinsed with a 12-mL portion of water, and the main product was eluted with a 21-mL portion of 2 M HCl. Water was removed by rotary evaporation and high vacuum. The sum of pools B and C was 0.013 g + 0.101 g = 0.114 g. The yield was 100%*

*0.147/375.48 = 0.00003915 moles; x 255.72 = 0.101 g.

H-1 (d_6_-DMSO)

14 (s, 1H), 8.63 (s, 3H), 6.87 (m, 1H), 6.73 (m, 1H), 4.18 (m, 1H), 2.85 (t, 2H), 2.64 (m, 1H), 1.00 (m, 4H).

C-13 (d_6_-DMSO)

170.40, 140.08, 133.12, 51.07, 31.97, 30.69, 5.18, 5.15.

HRMS calc. for C_8_H_14_NO_4_S (M+1) m/z 220.0644; found m/z 220.0629.

**Compound 23: 2-methylsulfonylacetophenone**

Phenacyl bromide (0.407 g, 2.04 mmol), sodium methanesulfinate (0.233 g, 0.228 mmol), and tetrabutylammonium bromide (0.0660g, 0.205 mmol) were stirred in acetonitrile (10 mL). The reaction was stirred overnight. After the solvent was removed by rotary evaporation, the product was extracted using 3 portions of ethyl acetate. After the combined organic layers were dried with Na_2_SO_4_, the solvent was removed with rotary evaporation. The crude product was eluted from silica with 50:50 ethyl acetate/hexanes. Removal of the solvent by rotary evaporation and high vacuum provided a white solid (0.240 g, 59.3% yield).

H-1 NMR (CDCl_3_)

8.04 (m, 2H), 7.76, m, 1H), 7.54 (m, 2H), 4.63 (s, 2H), 3.18 (s, 3H).

C-13 NMR (CDCl_3_)

189.23, 135.61, 134.77, 129.08, 129.27, 61.29, 41.83.

HRMS calc. for C_9_H_9_O_3_S (M-1) m/z 197.027242; found m/z 197.0286.

**Compound 24: (E)-4-(2-Morpholinocarbonyl)vinylpyridine**

**24A** Diethyl morpholinocarbonylmethylphosphonate

Triethyl phosphite (1.88 g, 13.3 mmol) and 2-chloroacetylmorpholine (2 g, 12.2 mmol) were heated to 125 °C with stirring until complete by P-31 NMR, about 16 hours. The crude reaction mixture was eluted from silica using 90:10 ethyl acetate/ethanol. Removal of the solvent by rotary evaporation and high vacuum provided the product (1.618 g, 57% yield).

H-1 NMR (CDCl_3_)

4.26-4.11 (m, 4H), 3.78-3.54 (m, 8H), 3.11 (d, J = 21.9, 2H), 1.36 (t, J = 6.9, 6H).

C-13 NMR (CDCl_3_)

163.38 (d, J = 5.6), 66.73, 62.76 (d, J = 6.6), 47.34, 42.41, 33.26 (d, J = 132.2), 16.37 (d, J = 6.2).

P-31 NMR (CDCl_3_)

20.86.

**24** 4-(2-Morpholinocarbonyl)vinylpyridine

Lithium chloride (0.184 g, 3.42 mmol) was suspended in acetonitrile (20 mL) with stirring at room temperature. Successively were added Diethyl morpholinocarbonylmethylphosphonate 24A (0.545 g, 2.06 mmol) in acetonitrile (0.8 mL), DBU (0.299 g, 1.87 mmol) in acetonitrile (0.7 mL), and 4-formylpyridine (0.199 g, 1.87 mmol) in acetonitrile (1.4 mL); the last solution was added dropwise. The reaction was stirred until the aldehyde was no longer visible by TLC, and the reaction was quenched with saturated NH_4_Cl (~5 mL). The volatiles were removed; a small volume of water was added; and the solution was extracted with 4 20-mL portions of DCM. The combined DCM layers were dried with MgSO_4_, and the solvent was removed by rotary evaporation. The product was eluted from silica using 95:5 acetone/methanol. The solvent was removed by rotary evaporation, and this partially purified product was repurified by eluting from fresh silica with 35:65 acetone/ethyl acetate. Removal of solvent by rotary evaporation and high vacuum provided the product (0.318 g, 77.9% yield).

H-1 NMR (CDCl_3_)

8.67 (m, 2H), 7.62 (d, J = 15.6, 1H), 7.42 (m, 2H), 7.05 (d, J = 15.6, 1H), 3.85-3.60 (m, 8H).

C-13 NMR (CDCl_3_)

164.44, 149.89, 143.03, 140.05, 121.90, 121.72, 66.78, 46.36, 42.59.

HRMS calc. for C_12_H_15_N_2_O_2_ (M+1) m/z 219.1134; found m/z 219.1144.

**Compound 25: (E)-4-Acrylamidopyridine**

LiCl (0.1086 g, 2.56 mmol) was suspended in ACN (10 mL) and stirred at room temperature. Successively were added diethyl carboxamidomethylphosphonate **26A** (0.2524, 1.29 mmol), DBU (0.170g, 1.12 mmol) in ACN (0.5 mL), and 4-formylpyridine (0.1167 g, 1.09 mmol) in ACN (0.5); the final solution was added dropwise. After two hours the reaction was quenched with a 3-mL portion of saturated NH_4_Cl. After the volatiles were removed with rotary evaporation, a small portion of water was added and this solution was extracted with 5 10-mL portions of DCM. The combined organic layers were dried with Na_2_SO_4_, and the solvent was removed with rotary evaporation. The crude product was eluted from silica with 15:85 to 20:80 ethanol/chloroform. Removal of the solvent via rotary evaporation and high vacuum provided a 0.033-g portion of a white, crystalline solid (21% yield).

H-1 (d_6_-DMSO)

8.620-8.600 (d, J = 6Hz, 2H), 7.665 (s, 1H), 7.529-7.508 (d, J = 6.3 Hz, 2H), 7.420-7.367 (m, J = 16 Hz, 1H), 7.274 (s, 1H), 6.856-6.802 (m, J = 16 Hz, 1H).

C-13 (d_6_-DMSO)

166.39, 150.81, 142.60, 137.11, 127.34, 122.14.

HRMS calc. for C_8_H_9_N_2_O (M+i) m/z 149.0715; found m/z 149.0709.

**Compound 26: (E)-2-(Carboxamido)vinylalanine hydrochloride**

**26A** Diethyl carboxamidomethylphosphonate

Triethyl phosphite (10.67 g, 64.2 mmol) and 2-Chloroacetamide (2.00 g, 21.4 mmol) were heated to approximately 110 °C for 12 hours. The crude product was recrystallized using 100 mL of 3:1 toluene/hexanes, and a second crop obtained to provide the product (2.523 g; 60.4% yield).

H-1 NMR (CDCl_3_)

6.80 (s, 1H), 5.67 (s, 1H), 4.18 (m, 4H), 2.88 (d, J = 20.7, 2H), 1.37 (t, J = 7.2, 6H).

C-13 NMR (CDCl_3_)

166.26 (d, J = 3), 62.87 (d, J = 7), 34.95 (d, J = 131), 16.32 (d, J = 6).

P-31 NMR (CDCl_3_)

22.54.

**26B** Boc-Ala(CH_2_CH=CHC(O)NH_2_)-OtBu

LiCl (0.0837 g, 1.97 mmol) was suspended in ACN (10 mL) and stirred at room temperature. Successively were added diethyl carboxamidomethylphosphonate **26A** (0.257 g, 1.317 mmol), DBU (0.167 g, 1.097 mmol) in ACN (0.7 mL), and Boc-Asp(H)-OtBu **14B** (0.300 g, 1.098 mmol) in ACN (0.7 mL); the final reagent was added dropwise. The reaction was stirred for 2 hrs and quenched with saturated NH_4_Cl (3 mL). After the volatiles were removed, a small portion of water was added, and this solution was extracted with 4 10-mL portions of DCM and one 10-mL portion of EtOAc. The combined organic layers were extracted against brine (5 mL), dried with Na_2_SO_4_, and the solvent removed by rotary evaporation. The crude product was eluted from silica using 75:25 EtOAc/hexanes. Removal of the solvent by rotary evaporation and high vacuum provided a 0.238-gram portion of the product (72.2% yield).

H-1 NMR (CDCl_3_)

6.80-6.65 (m, 1H), 5.92 (d, J = 15, 1H), 5.47 (broad s, 2H), 5.166 (d, J = 7.5, 1H), 4.40-4.25 (m, 1H), 2.79-2.52 (m, 2H), 1.49 (s, 9H), 1.46 (s, 9H).

C-13 NMR (CDCl_3_)

170.46, 167.05, 155.16, 139.69, 126.03, 82.60, 79.97, 53.03, 35.57, 28.31, 28.02.

**26** (E)-2-(Carboxamido)vinylalanine hydrochloride

The protected amino acid **26B** (0.238 g, 0.757 mmol) was dissolved in DCM (0.6 mL) and stirred at 0 °C. TFA (2.4 mL) was added dropwise, and the reaction stirred. The reaction was followed to completion by extracting small portions of the reaction mixture, with EtOAc/NaHCO_3_, spotting the EtOAc layer onto TLC plates, and staining with PMA or KMnO_4_. The solvent was removed by rotary evaporation and high vacuum to provide the product (0.107 g; 72.7% yield).

H-1 NMR (D_2_O)

6.735-6.633 (m, 1H), 6.189 (d, *J* = 15.3 Hz, 1H), 4.222 (q, *J* = 6.1 Hz, 1H) 2.974-2.815 (m, 2H).

C-13 NMR (D_2_O)

171.28, 169.90, 137.69, 127.52, 52.09, 32.33

HRMS calc. for C_6_H_11_N_2_O_3_ (M+1) m/z 159.0770; found m/z 159.0701.

**Compound 27: (E)-2-(N,N-Dimethylcarboxamido)vinylalanine hydrochloride**

**27A** Diethyl N,N-dimethylcarboxamidomethylphosphonate

Dimethyl chloroacetamide (1.52 g, 12.3 mmol) and triethylphosphite (1.90 g, 11.4 mmol) were heated to 125 °C with stirring for 17 hours. The crude product was purified over 64 grams of silica using 92:8 to 90:10 ethyl acetate/methanol. Rotary evaporation and high vacuum yielded the product (0.84 grams, 33% yield).

H-1 NMR

4.19 (m, 4H), 3.14 (s, 3H), 3.07 (d, J = 22.2, 2H), 2.99 (d, J = 1.5, 3H), 1.35 (t, J = 7.2, 6H).

C-13 NMR

164.88 (d, J = 6.0), 62.62 (d, J = 6.3 Hz), 38.58 (s), 35.85 (s), 33.40 (d, J = 153.2), 16.37 (d, J = 6.5).

P-31 NMR

21.35 (s)

**27B** Boc-Ala(CH_2_CH=CHC(O)C_2_H_5_)-OtBu

Lithium chloride (0.111 g, 2.62 mmol) was suspended in acetonitrile (5 mL) at 25 °C. Successively were added Diethyl N,N-dimethylcarboxyamidomethylphosphonate **27A** (0.298 g, 1.33 mmol) in acetonitrile (1 mL), DBU (0.203 g, 1.29 mmol) in acetonitrile (1 mL), and Boc-Asp(H)-OtBu **14B** (0.340 g, 1.25 mmol) in acetonitrile (0.8 mL); the last solution was added dropwise. The reaction was stirred for 4 hours then quenched with saturated NH_4_Cl. After the volatiles were removed and a small portion of water added, the solution was extracted with four 10-mL portions of DCM. After the combined organic layers were dried with Na_2_SO_4_, the solvent was removed by rotary evaporation. The product was eluted from silica with 60:40 ethyl acetate/hexanes to 100% ethyl acetate. Removal of the solvent by rotary evaporation and high vacuum provided the product (0.201 g, 49% yield).

H-1

6.74 (dt, J = 15.0, 7.5, 1H), 6.34 (d, J = 15, 1H), 5.11 (d, J = 8.1, 1H), 4.38-4.28 (m, 1H), 3.04 (s, 6H), 2.79-2.56 (m, 2H), 1.47 (s, 9H), 1.44 (s, 9H).

C-13

170.64, 166.16, 155.09, 139.38, 123.70, 82.43, 79.82, 53.08, 36.4 (broad) 35.40, 28.34, 28.04.

**27** (E)-2-(N,N-Dimethylcarboxamido)vinylalanine hydrochloride

The protected amino acid **27B** (0.201 g, 0.612 mmol) was stirred in DCM (0.5 mL) at 0°C. TFA (2 mL) was added dropwise. The cooling bath was removed and the reaction was allowed to stir overnight. The volatiles were removed with rotary evaporation, addition of toluene followed by rotary evaporation, and high vacuum. A 7-mL column of Dowex-50(H^+^) was prepared, and the sample dissolved in water (4.5 mL) and the pH was checked. After the solution had been applied to the column, it was rinsed with water (10 mL), and the load and wash were set aside. The product was eluted with 1 M HCl (25 mL) and 2 M HCl (25 mL). Rotary evaporation and high vacuum provided the product (0.130 g, 95.4% yield).

H-1 NMR (d_6_-DMSO)

8.50 (s, 3H), 6.58 (m, 2H), 4.10 (m, 1H), 3.04 (s, 3H), 2.87 (s, 3H), 2.81-2.67 (m, 2H).

C-13 NMR (d_6_-DMSO)

170.69, 165.33, 137.20, 125.83, 51.60, 37.27, 35.53, 32.88.

HRMS calc. for C_8_H_15_O_3_N_2_ (M+1) m/z 187.1083; found m/z 187.1096.

**2. Compound purity assessment (LC-MS Chromatograms)**

Compound 1: (E)-1,4-Dihydroxy-2-(methylsulfonyl)vinylbenzene

Negative total ion chromatogram


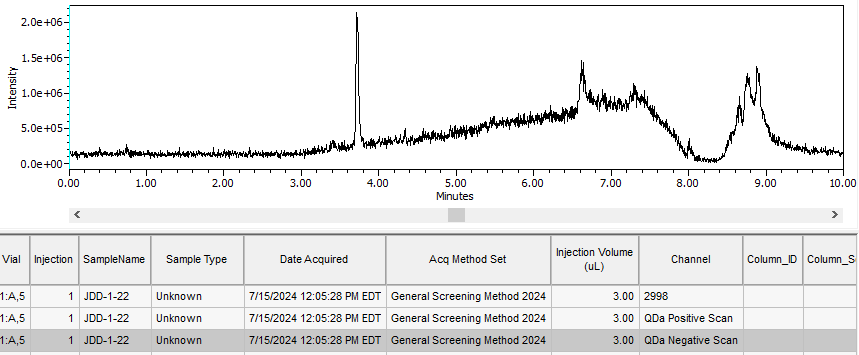


Compound 2: (E)-4-(2-Methylsulfonyl)vinylacetanilide

Positive total ion chromatogram


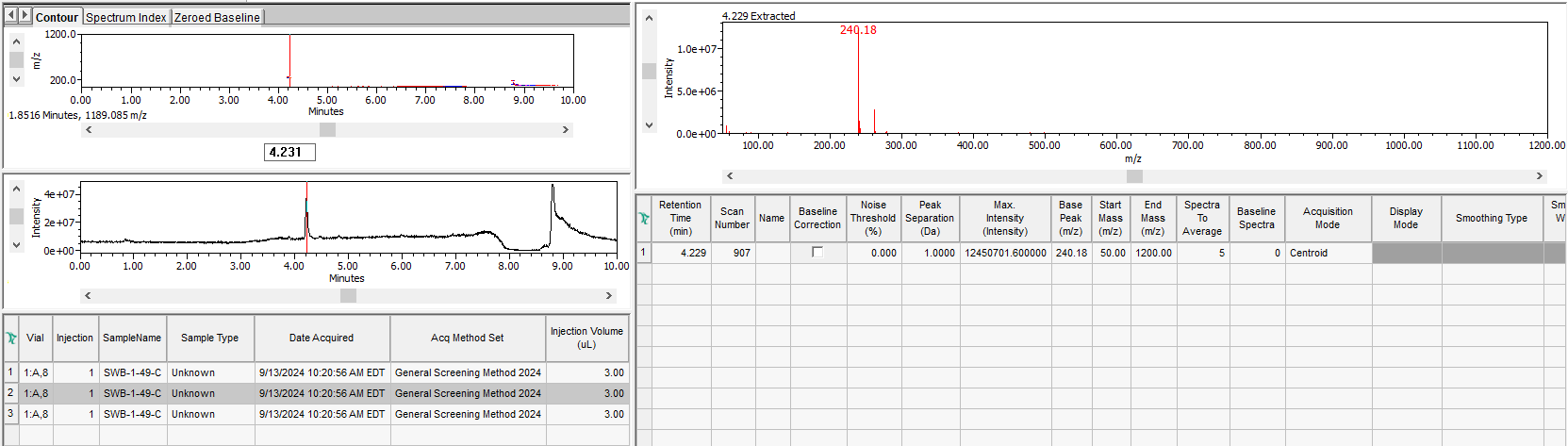


Compound 3: (E)-4-(2-Methylsulfonyl)vinylbenzamide

Positive total ion chromatogram


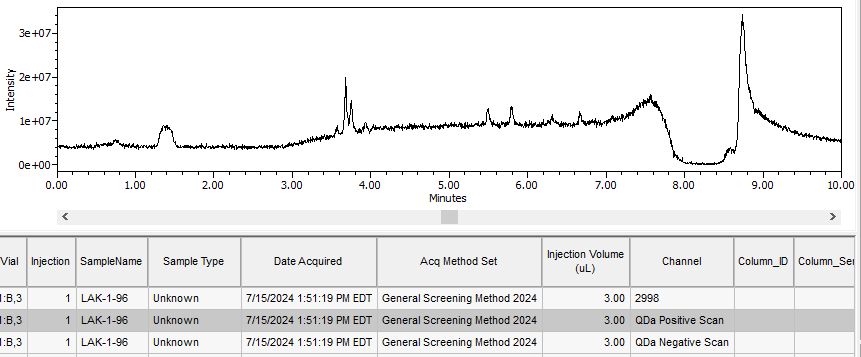


Compound 4: 5-Hydroxy-2-(2-methylsulfonyl)vinylpyridine

Negative total ion chromatogram


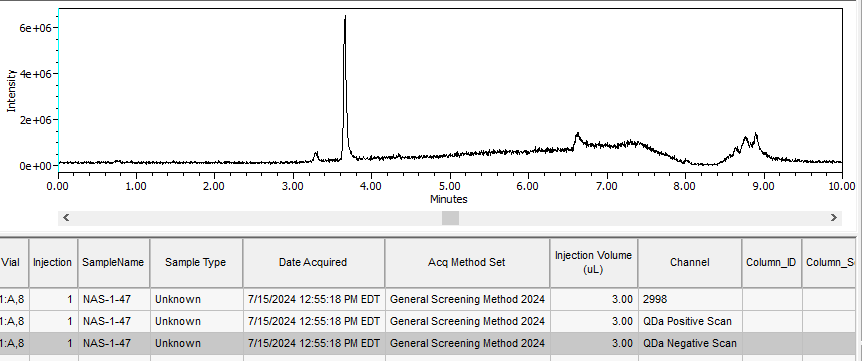


Compound 5: (E)-2-(2-Methylsulfonyl)vinyl-5-nitrothiophene

Negative total ion chromatogram


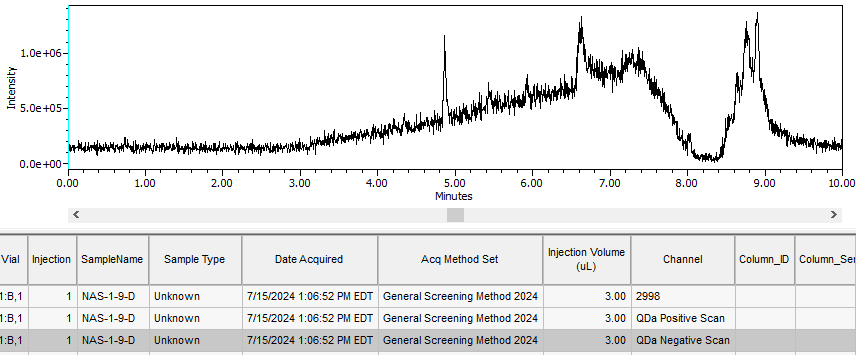


Compound 6: (E)-4-(2-Methylsulfonyl)vinylquinoline

Positive total ion chromatogram


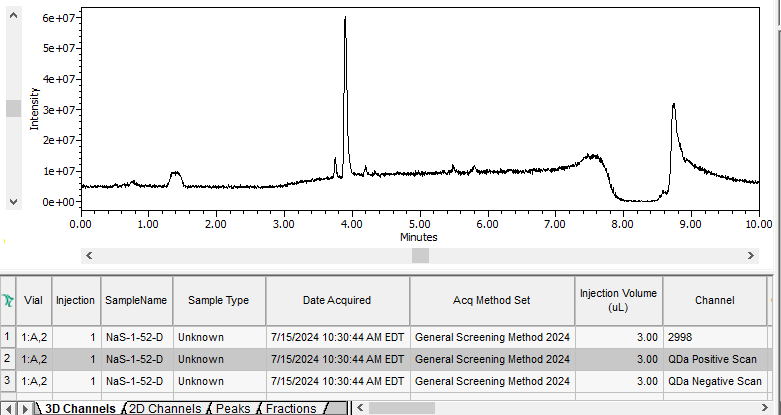


Compound 7: (E)-2-(2-Methylsulfonyl)vinylquinoline

Positive total ion chromatogram


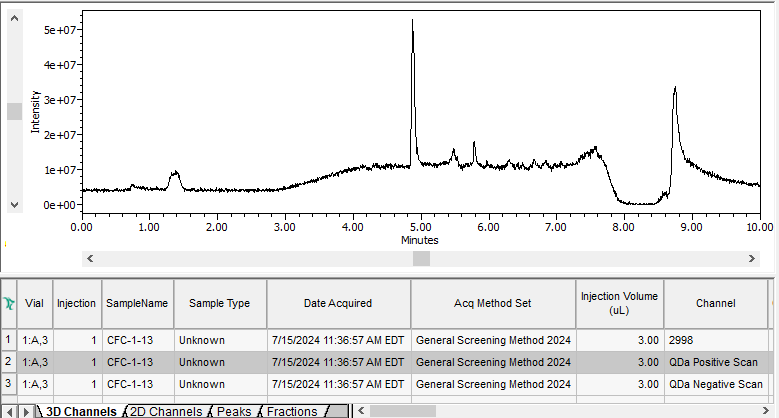


Compound 8: (E)-5-(2-Methylsulfonyl)vinylisoquinoline

Positive total ion chromatogram


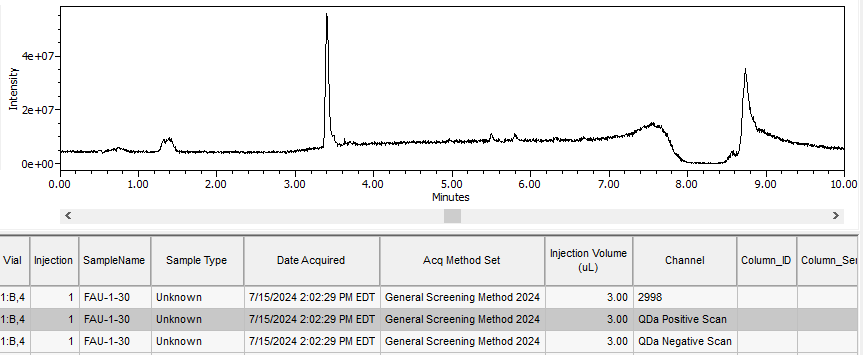


Compound 9: (E)-5-(2-Cyclopropylsulfonyl)vinylisoquinoline

Positive total ion chromatogram


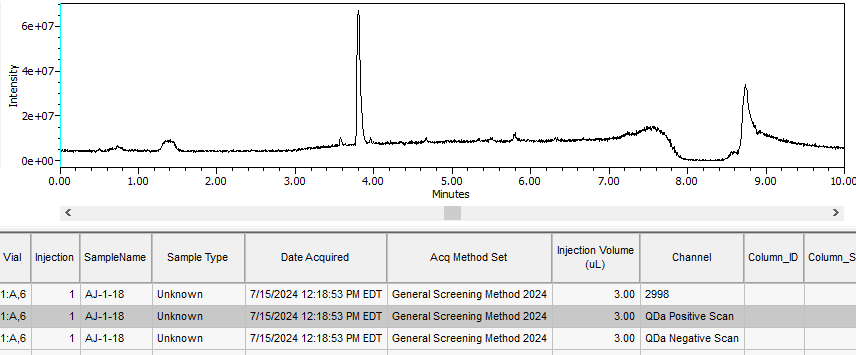


Compound 11: (E)-4-(2-morpholinosulfonyl)vinylpyridine

Positive total ion chromatogram


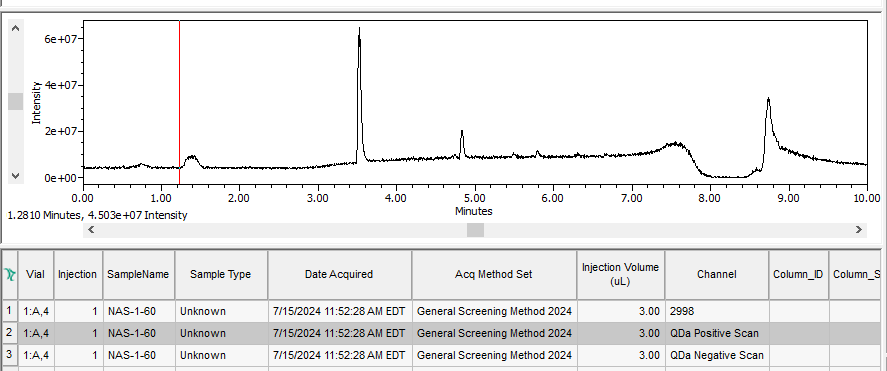


Compound 12: 3-Methylsulfonylacrylamide

Negative total ion chromatogram


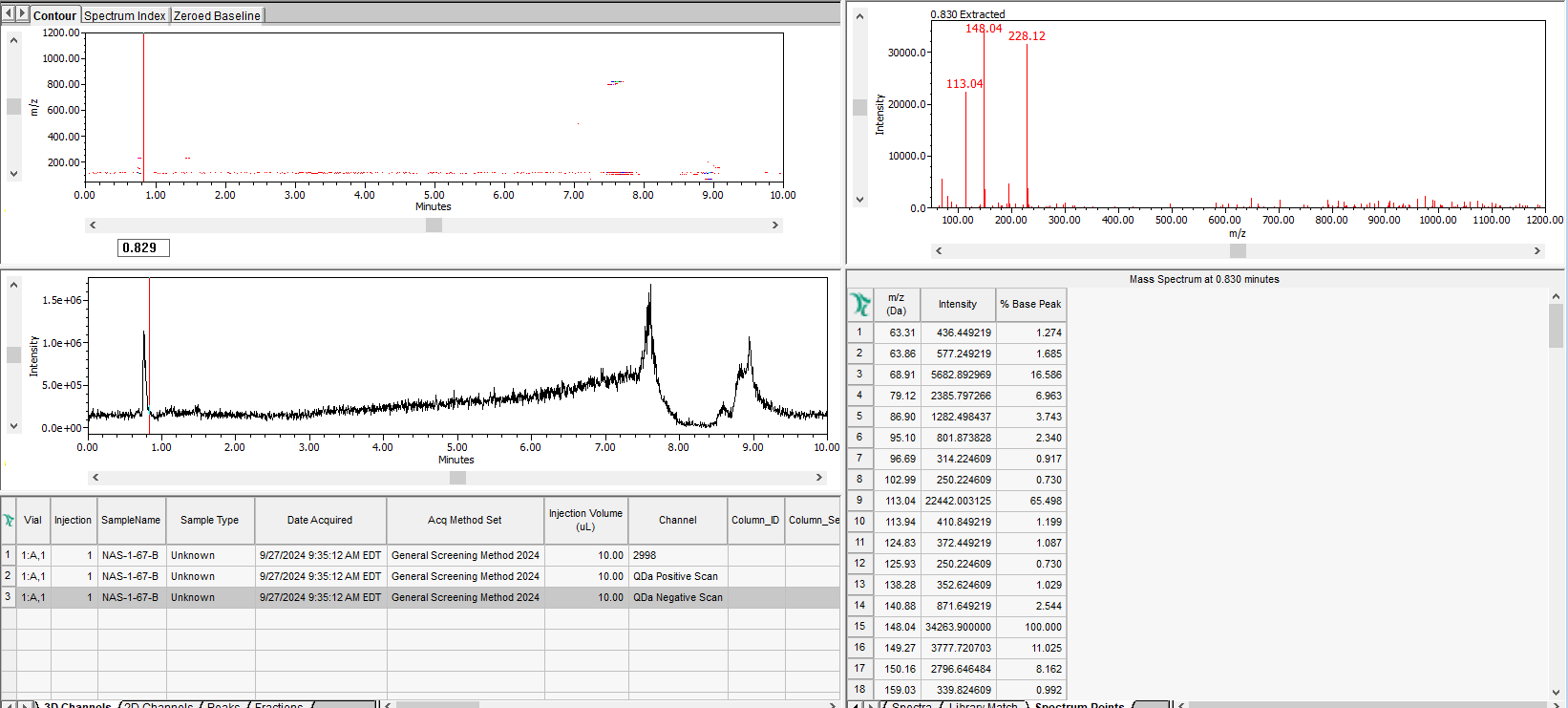


Compound 13: 3-Phenylsulfonylacrylamide

Positive total ion chromatogram


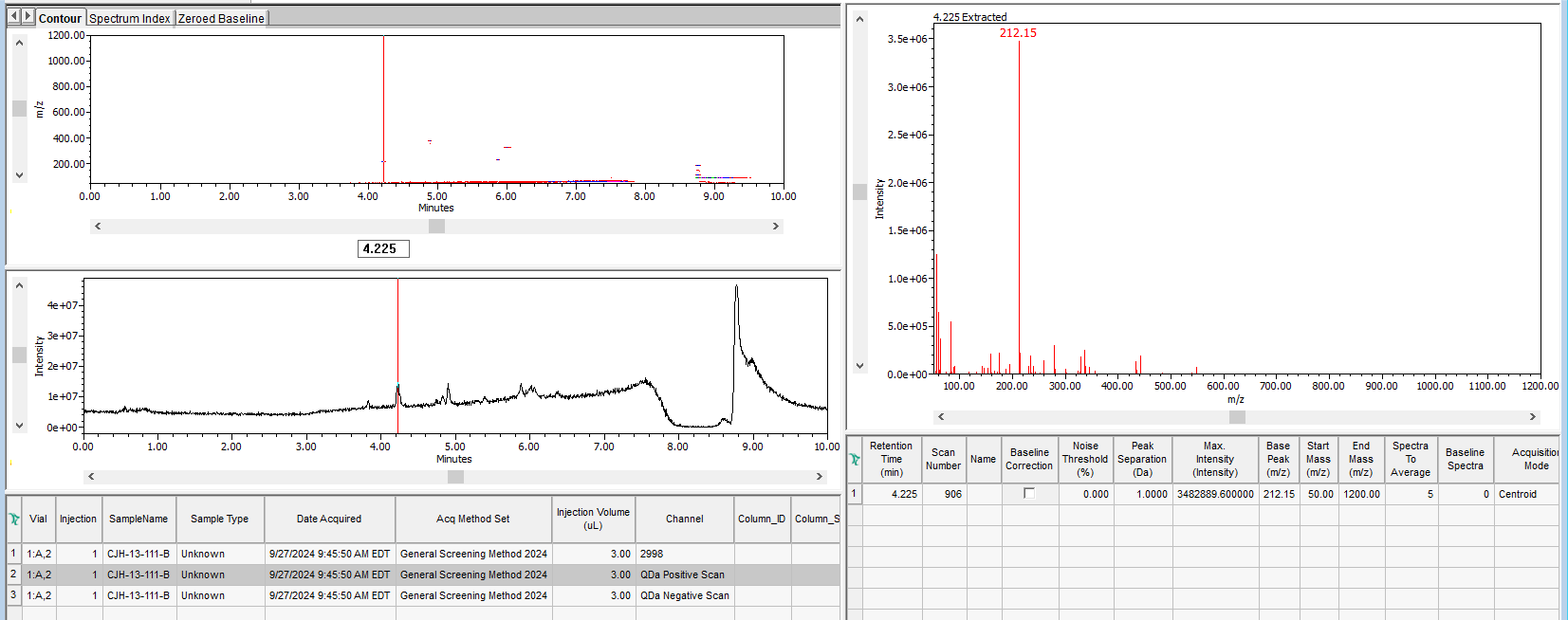


Compound 14: (E)-2-(Methylsulfonyl)vinylalanine hydrochloride

Positive total ion chromatogram


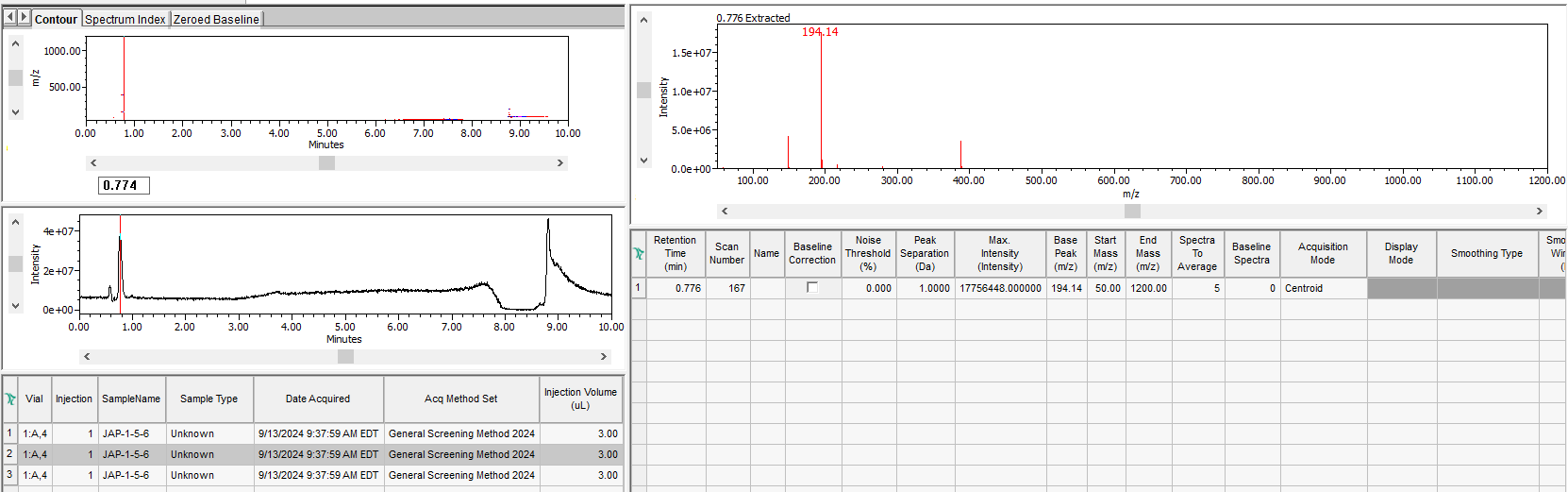


Compound 15: (E)-2-(N,N-Dimethylsulfonamido)vinylalanine hydrochloride

Positive total ion chromatogram


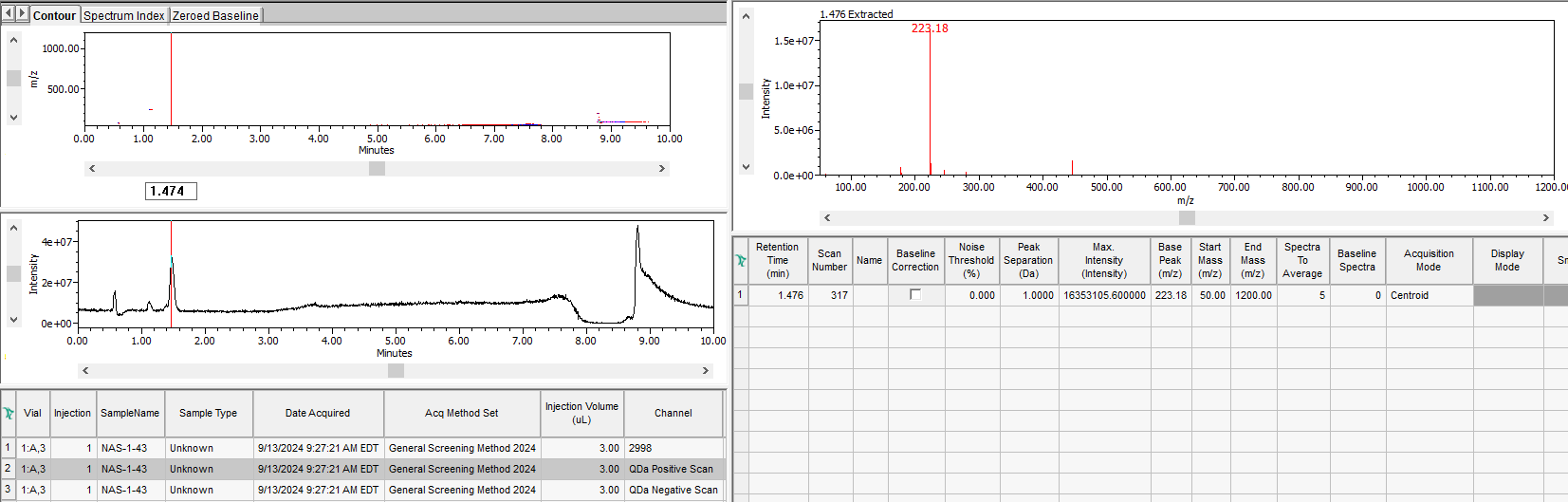


Compound 16: (E)-2-(Benzylsulfonyl)vinylalanine hydrochloride

Positive total ion chromatogram


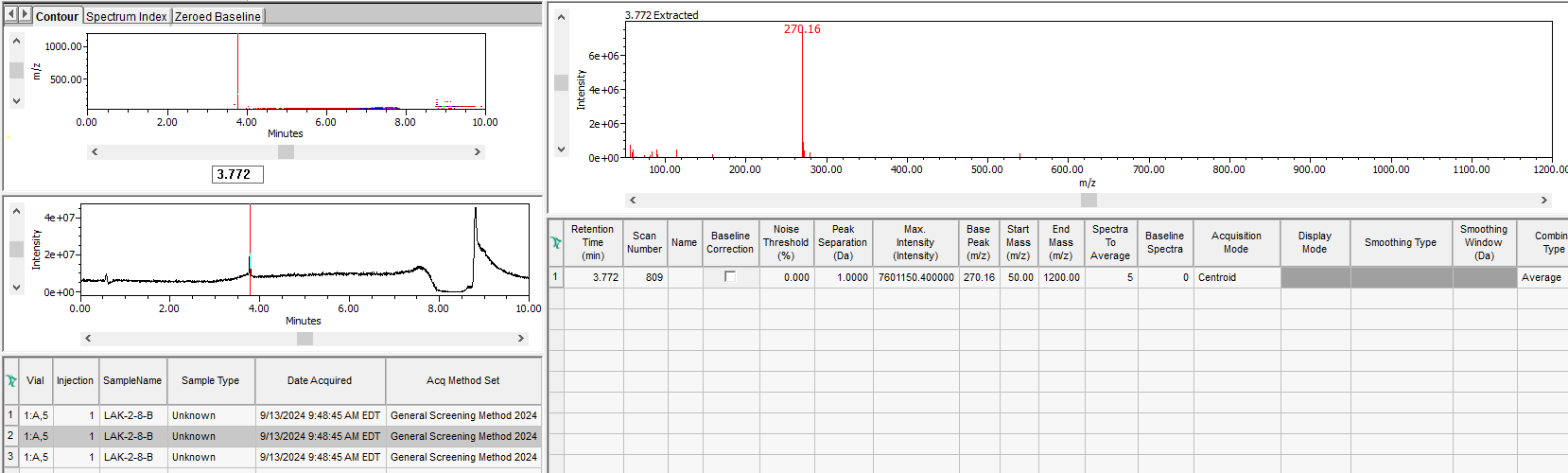


Compound 17: (E)-2-(Isopropylsulfonyl)vinylalanine hydrochloride

Positive total ion chromatogram


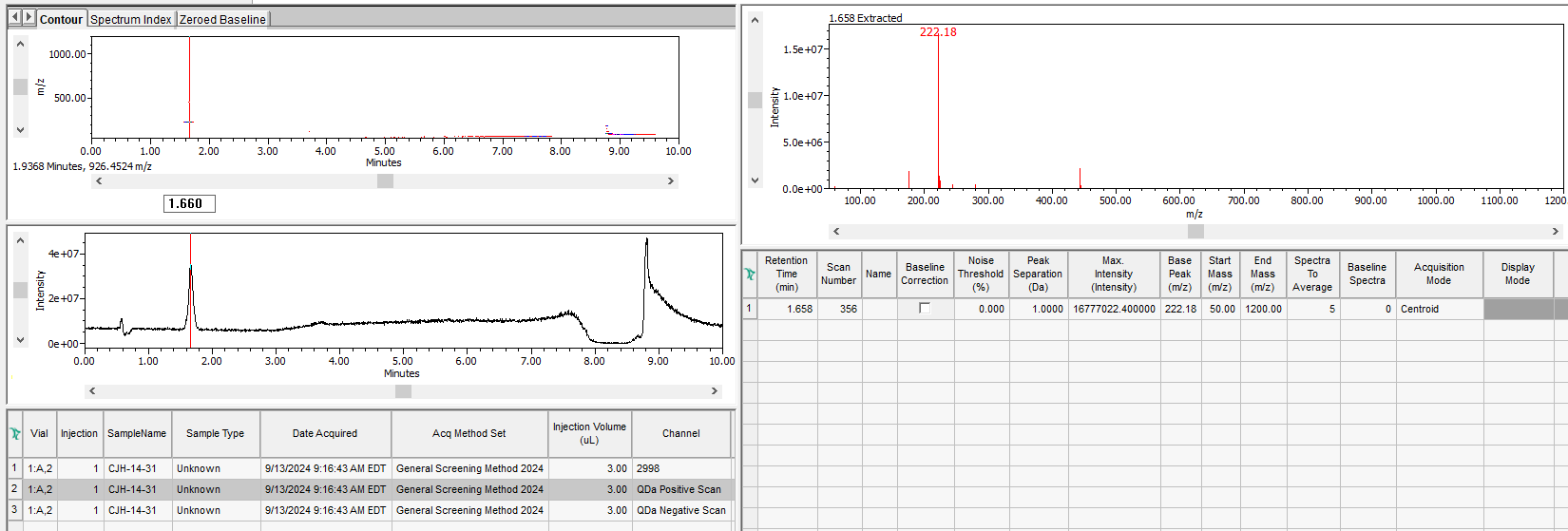


Compound 18: (E)-2-(Cyclopropylsulfonyl)vinylalanine hydrochloride

Positive total ion chromatogram


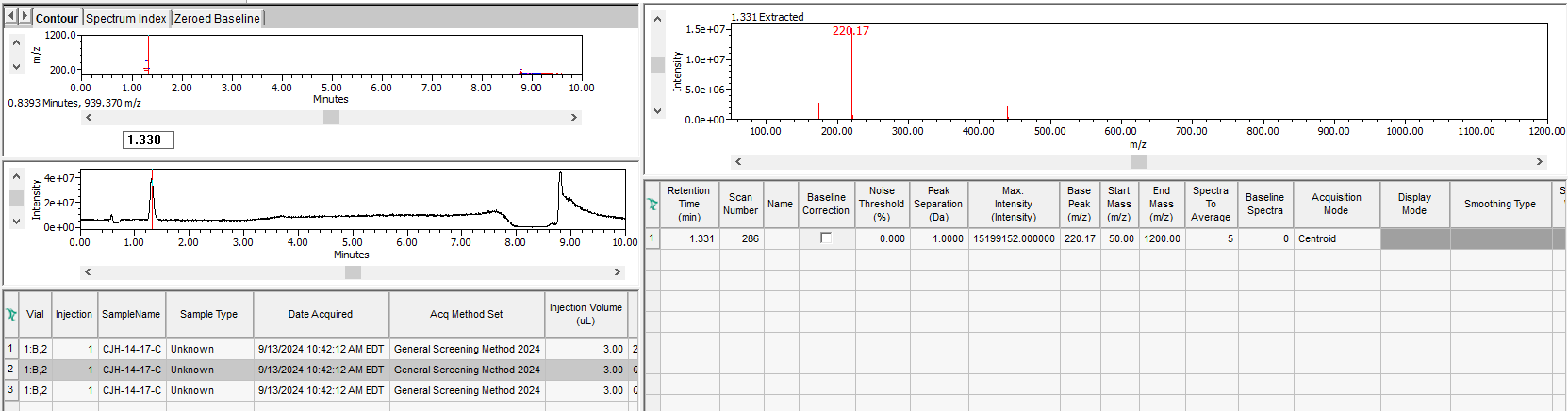


Compound 23: 2-Methylsulfonylacetophenone

Negative total ion chromatogram


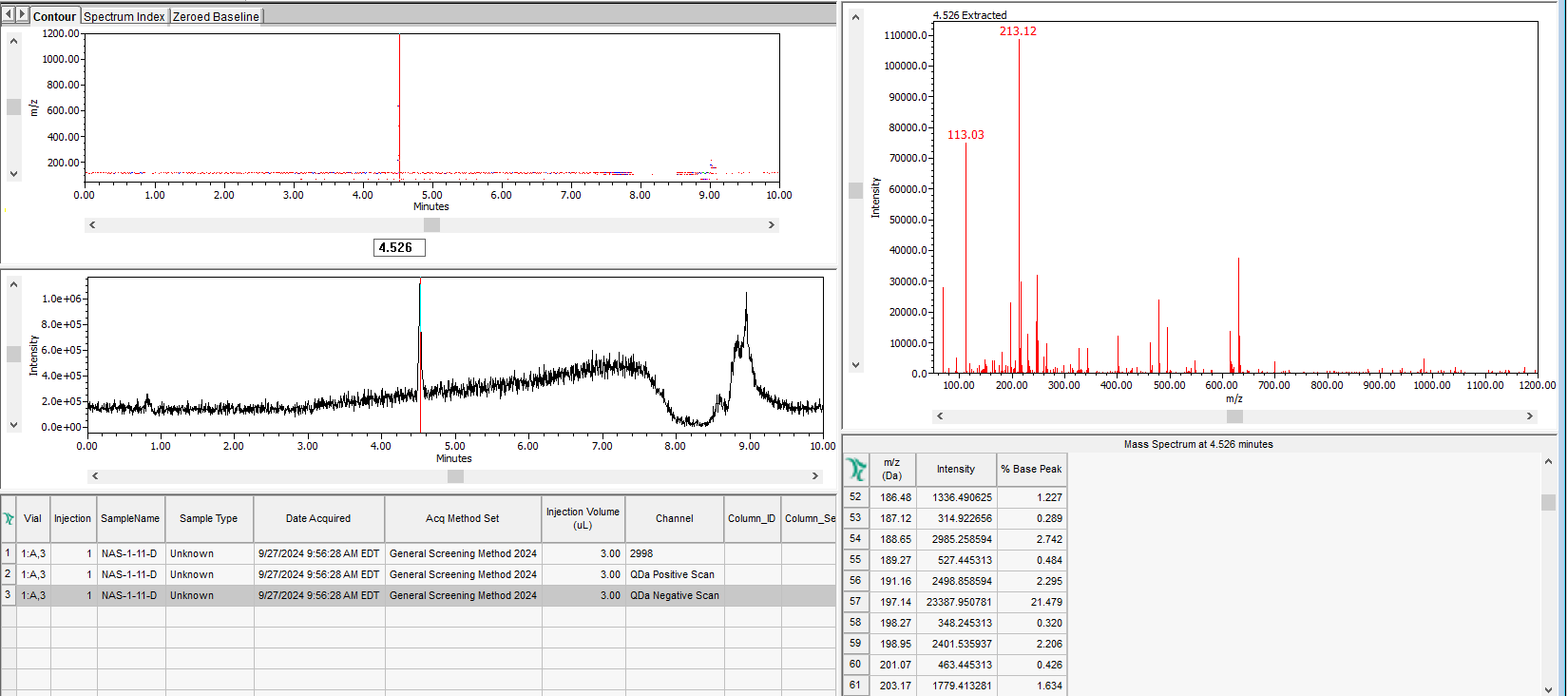


Compound 24: (E)-4-(2-Morpholinocarbonyl)vinylpyridine

Positive total ion chromatogram


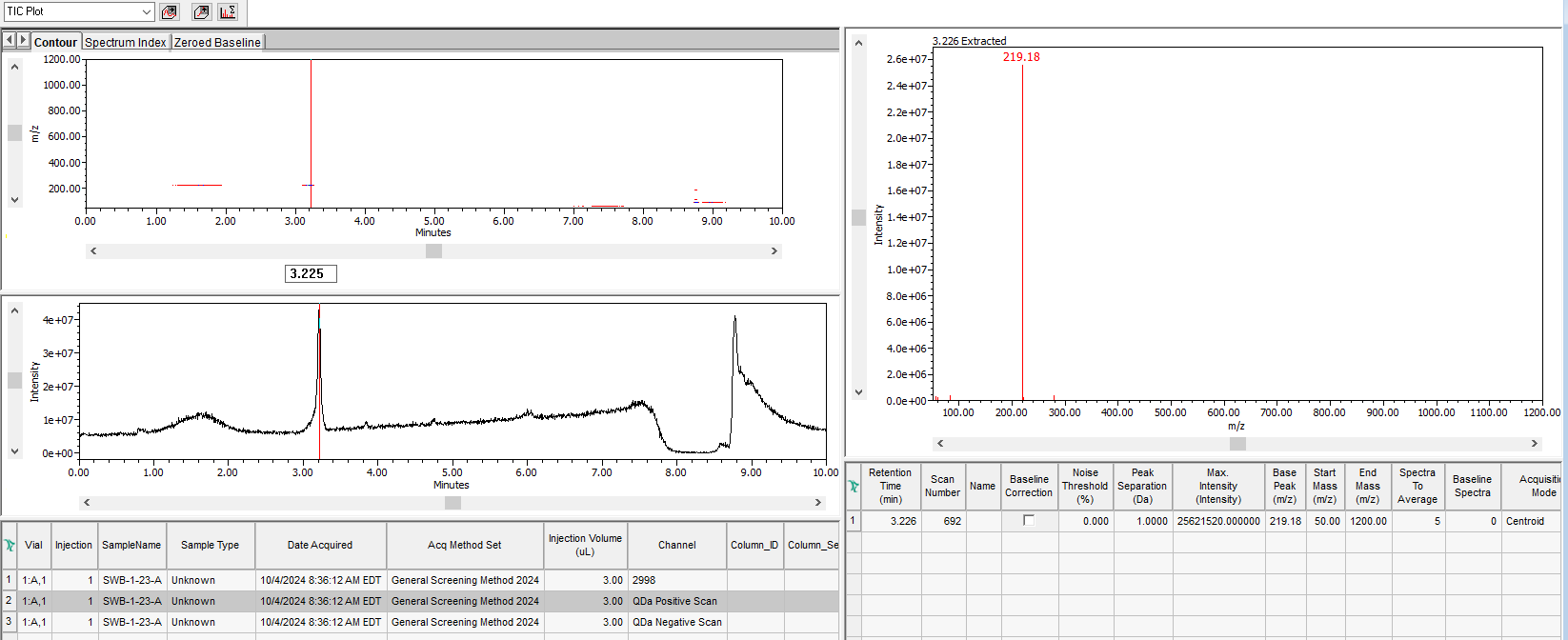


Compound 25: (E)-4-Acrylamidopyridine

Positive total ion chromatogram


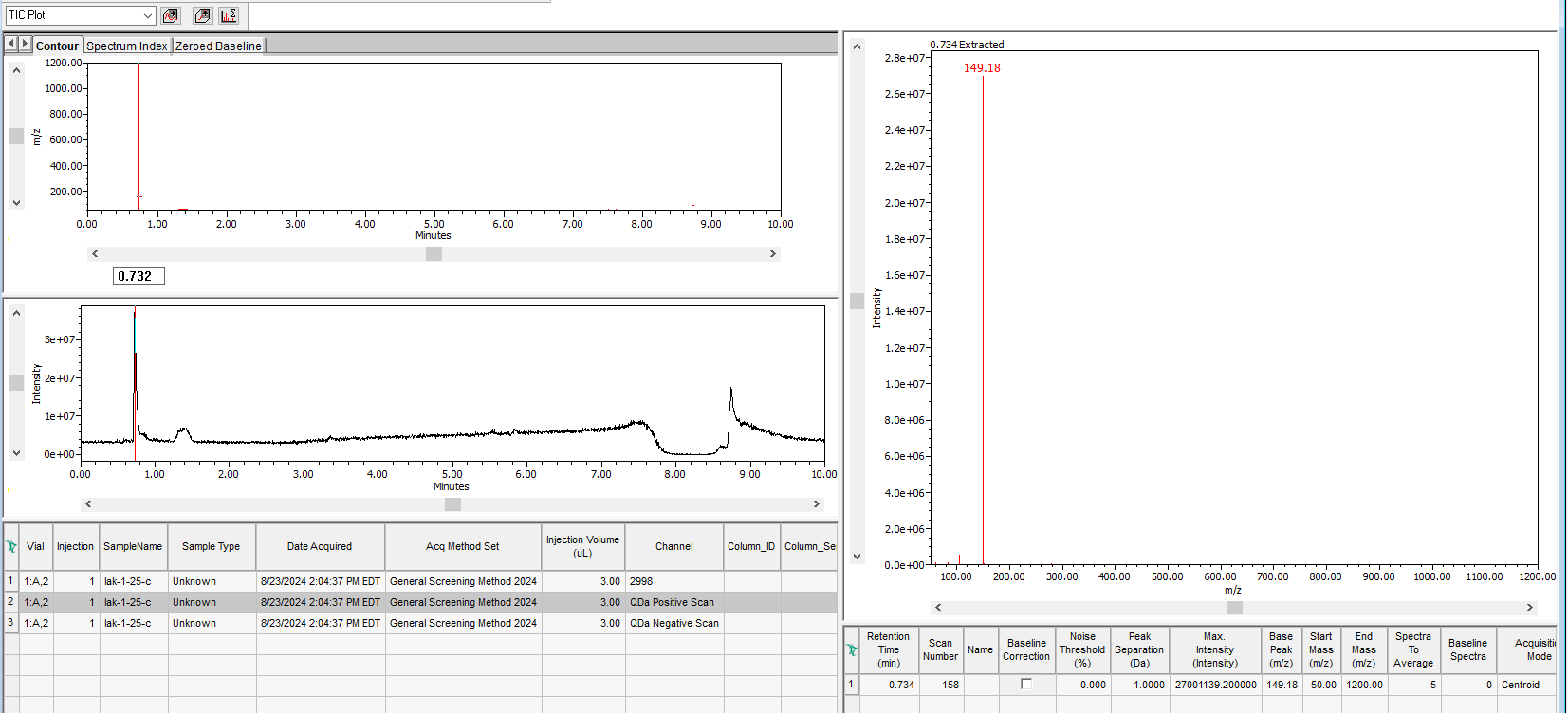


Compound 26: (E)-2-(Carboxamido)vinylalanine hydrochloride

Positive total ion chromatogram


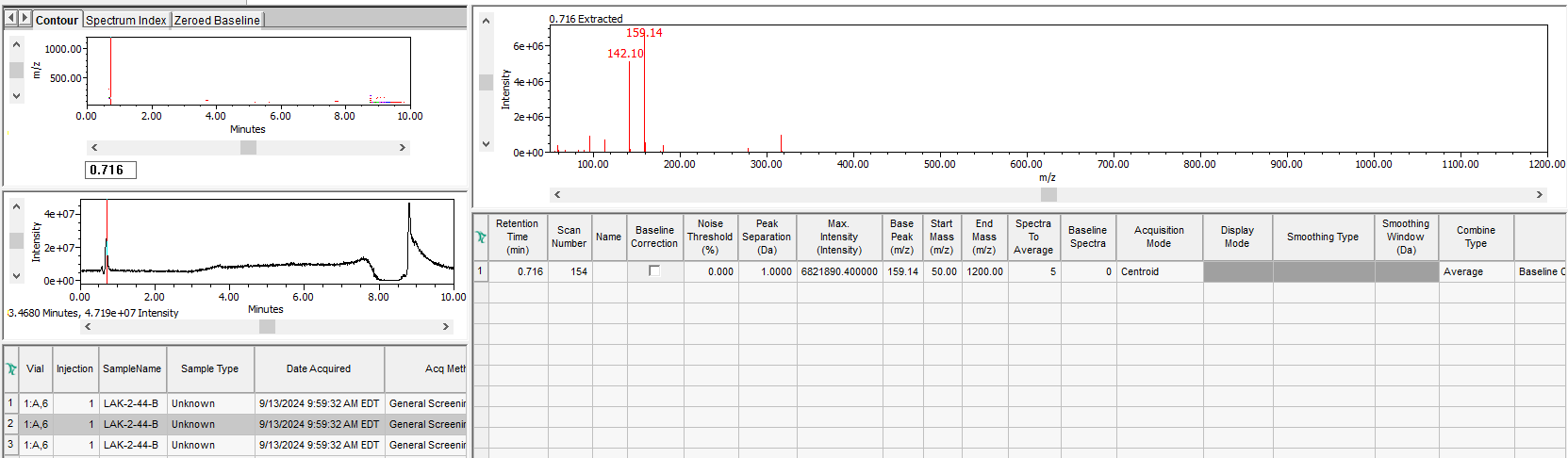


Compound 27: (E)-2-(N,N-Dimethylcarboxamido)vinylalanine hydrochloride

Positive total ion chromatogram


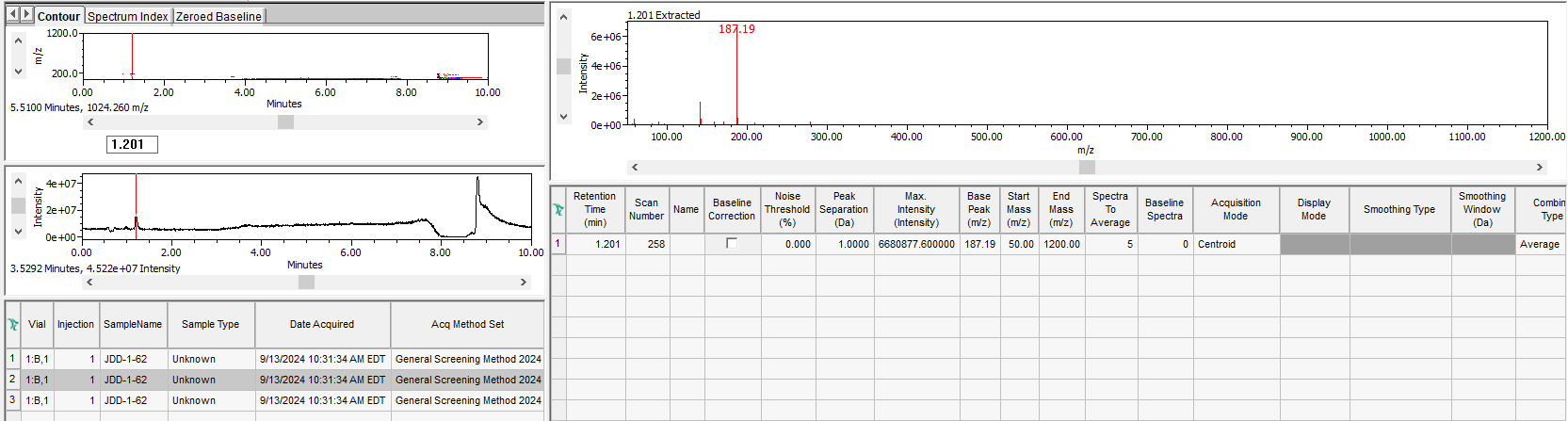


**3. NMR Spectra**

**1.1** **(E)-1,4-Dihydroxy-2-(2-methylsulfonyl)vinylbenzene**

**1.2 (E)-4-(2-Methylsulfonyl)vinylacetanilide**

**1.3 (E)-4-(2-Methylsulfonyl)vinylbenzamide**

**1.4 (E)-5-Hydroxy-2-(2-methylsulfonyl)vinylpyridine**

**1.5 (E)-2-(2-Methylsulfonyl)vinyl-5-nitrothiophene**

**1.6 (E)-4-(2-Methylsulfonyl)vinylquinoline**

**1.7 (E)-2-(2-Methylsulfonyl)vinylquinoline**

**1.8 (E)-5-(2-Methylsulfonyl)vinylisoquinoline**

**1.9 (E)-5-(2-Cyclopropylsulfonyl)vinylisoquinoline**

**1.10 4-(2-pyridylsulfonyl)vinylpyridine**

**1.11 (E)-4-(2-Morpholinosulfonyl)vinylpyridine**

**1.12 3-Methylsulfonylacrylamide**

**1.13 3-Phenylsulfonylacrylamide**

**1.14 (E)-2-(Methylsulfonyl)vinylalanine hydrochloride**

**1.15 (E)-2-(N,N-dimethylsulfonamido)vinylalanine hydrochloride**

**1.16 (E)-2-(Benzylsulfonyl)vinylalanine hydrochloride**

**1.17 (E)-2-(Isopropylsulfonyl)vinylalanine hydrochloride**

**1.18 (E)-2-(Cyclopropylsulfonyl)vinylalanine hydrochloride**

**1.23 2-methylsulfonylacetophenone**

**1.24 (E)-4-(2-Morpholinocarbonyl)vinylpyridine**

**1.25 (E)-4-Acrylamidopyridine**

**1.26 (E)-2-(Carboxamido)vinylalanine hydrochloride**

**1.27 (E)-2-(N,N-Dimethylcarboxamido)vinylalanine hydrochloride**

**4. Stereochemical analysis of MTPA-(2-propylsulfony)lvinylalanine**

1-Benzotriazol-1-yl-3,3,3-trifluoro-2-methoxy-2-phenylpropan-1-one ((R-MTPA-Bt,) was prepared as described (Katritzki 2007) with minor modifications. Thionyl chloride was distilled from quinoline. MTPA-Bt (19 µmol, 6.5 mg), 2-propylsulfonylvinylalanine hydrochloride, cpd. 17 (19 µmol, 5.0 mg), and triethylamine (49 µmol, 6.7 µL) were combined in 2:1 acetonitrile/H_2_O and stirred for 30 hr. Volatiles were removed by rotary evaporation, and the residue was partitioned between ethyl acetate (1.6 mL) and 4 M HCl (0.33 mL). The ethyl acetate layer was dried with Na_2_SO_4_, filtered, and the solvent was removed with rotary evaporation and high vacuum. The yield was 7.7 mg (92%). The epimeric ratio was 9:1.

*Reference*

Katritzki AR et al., J. Org. Chem, Vol. 72, No. 11, 2007 4271. 10.1021/jo070278a.

**5. Microbiology**

**Table S1: Disk diffusion**

| Compound Number | Zone of inhibition^1^ |
| --- | --- |
| 1 | 6 |
| 2 | 6 |
| 3 | 6 |
| 4 | 6 |
| 5 | 6.2 |
| 6 | 6.5 (hazy) |
| 7 | 11 (hazy) |
| 8 | 6 |
| 9 | 6 |
| 10 | 6 |
| 11 | 6 |
| 12 | 6 |
| 13 | 6 |
| 14 | 6 |
| 15 | 6 |
| 16 | 6 |
| 17 | Not determined |
| 18 | 6 |
| 19 | 6 |
| 20 | 6 |
| 21 | 6 |
| 22 | 6 |
| 23 | 6 |
| 24 | 6 |
| 25 | 6 |
| 26 | 6 |
| 27 | 6 |

^1^A ZOI of 6 mm indicates no inhibition.
